# Supplementary figures and images for: Inhibition of Kelch-like epichlorohydrin-related protein 1 promotes the progression and drug resistance of lung adenocarcinoma
Source: PeerJ. 2021 Aug 19;9:e11908. doi: 10.7717/peerj.11908 (PMC8380428; doi:10.7717/peerj.11908)

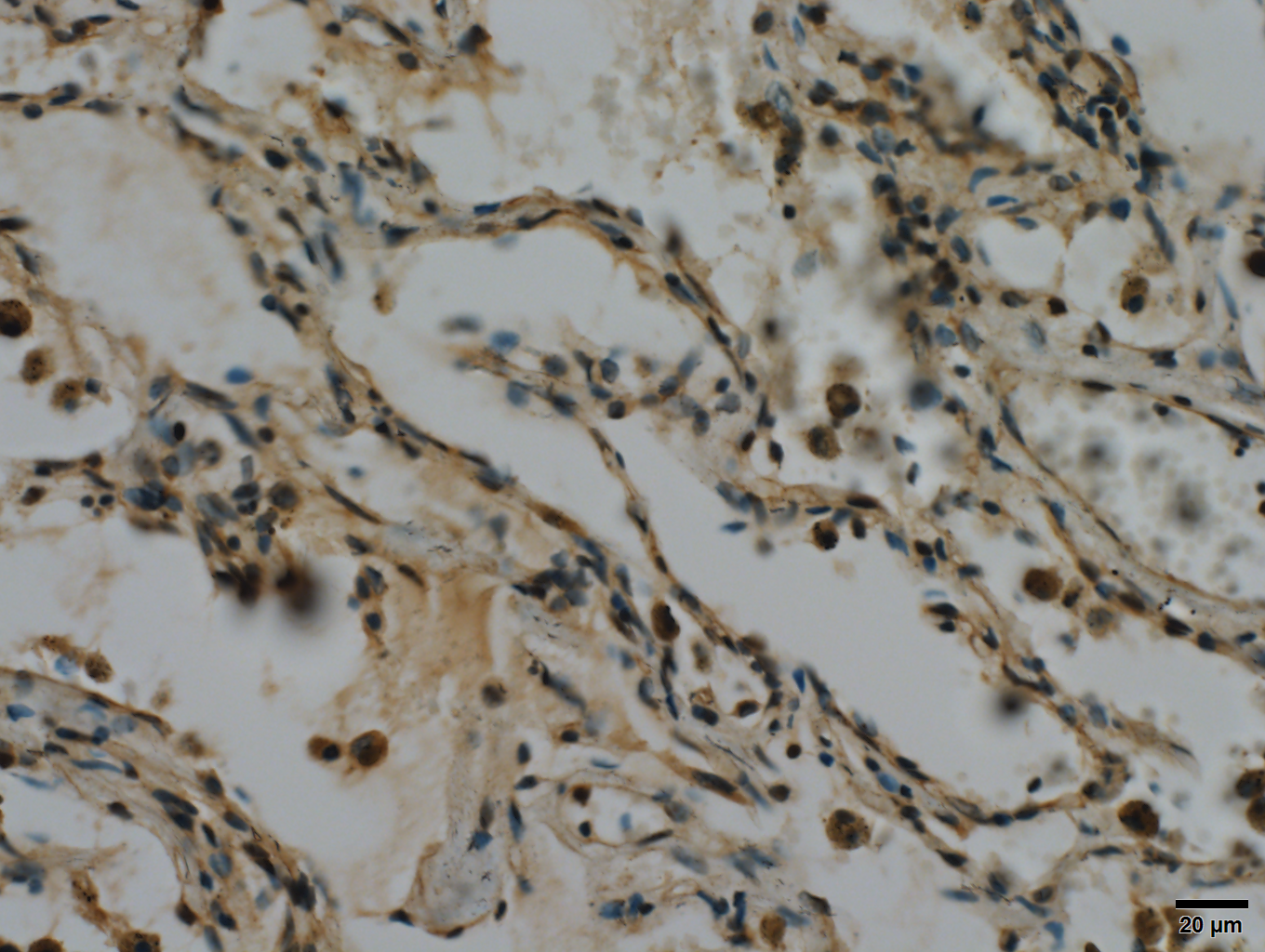

Supplement: Supplemental Information 2 — The immunohistochemistry assay and Keap1 mRNA in different lung cancer cell lines and HBE by RT-qPCR in Fig. 1. [file peerj-09-11908-s002.zip › Figure1 raw data/Immunohistochemistry (IHC) assay/Normal.tif]

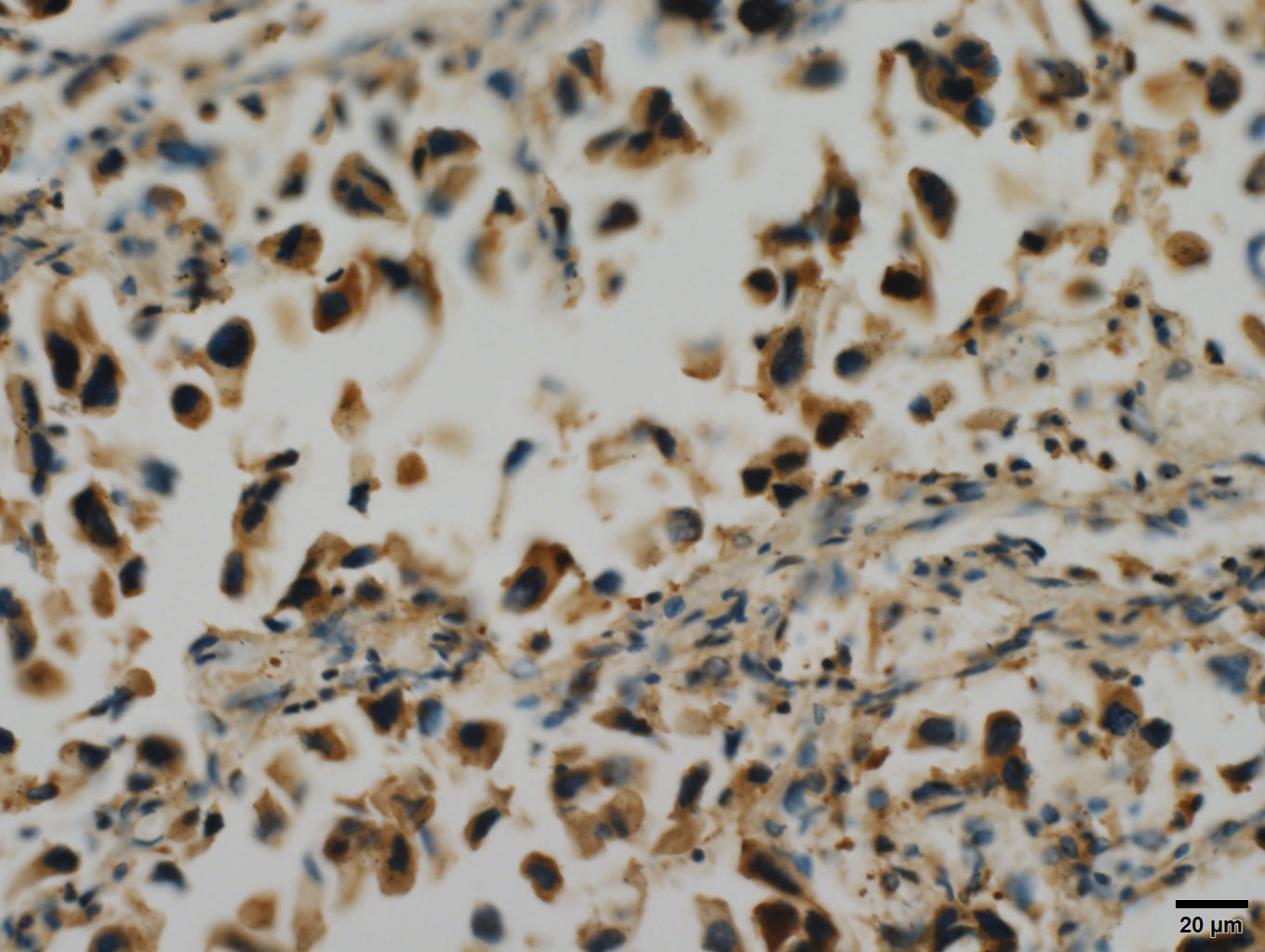

Supplement: Supplemental Information 2 — The immunohistochemistry assay and Keap1 mRNA in different lung cancer cell lines and HBE by RT-qPCR in Fig. 1. [file peerj-09-11908-s002.zip › Figure1 raw data/Immunohistochemistry (IHC) assay/StageI.tif]

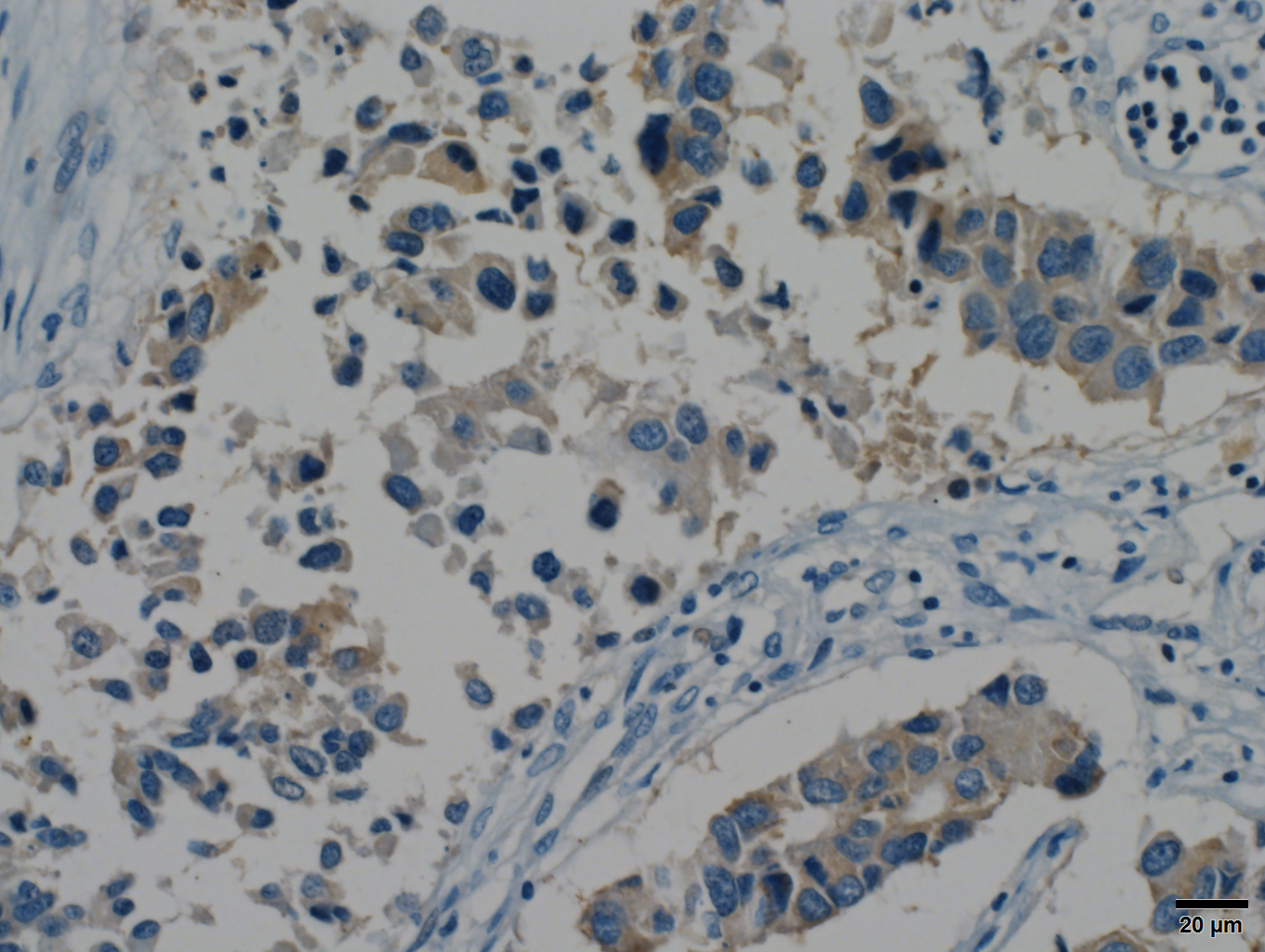

Supplement: Supplemental Information 2 — The immunohistochemistry assay and Keap1 mRNA in different lung cancer cell lines and HBE by RT-qPCR in Fig. 1. [file peerj-09-11908-s002.zip › Figure1 raw data/Immunohistochemistry (IHC) assay/StageII.tif]

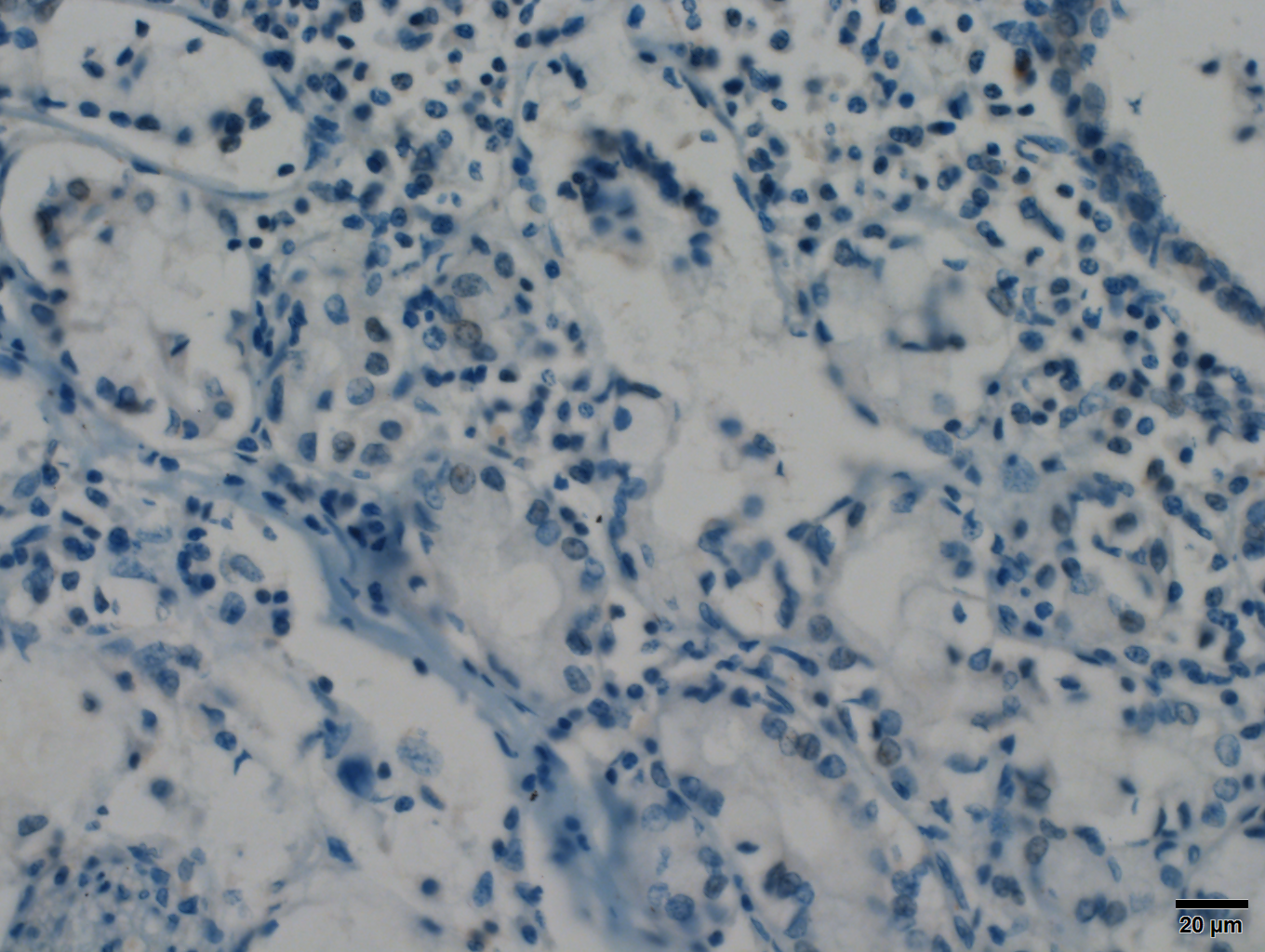

Supplement: Supplemental Information 2 — The immunohistochemistry assay and Keap1 mRNA in different lung cancer cell lines and HBE by RT-qPCR in Fig. 1. [file peerj-09-11908-s002.zip › Figure1 raw data/Immunohistochemistry (IHC) assay/StageIII.tif]

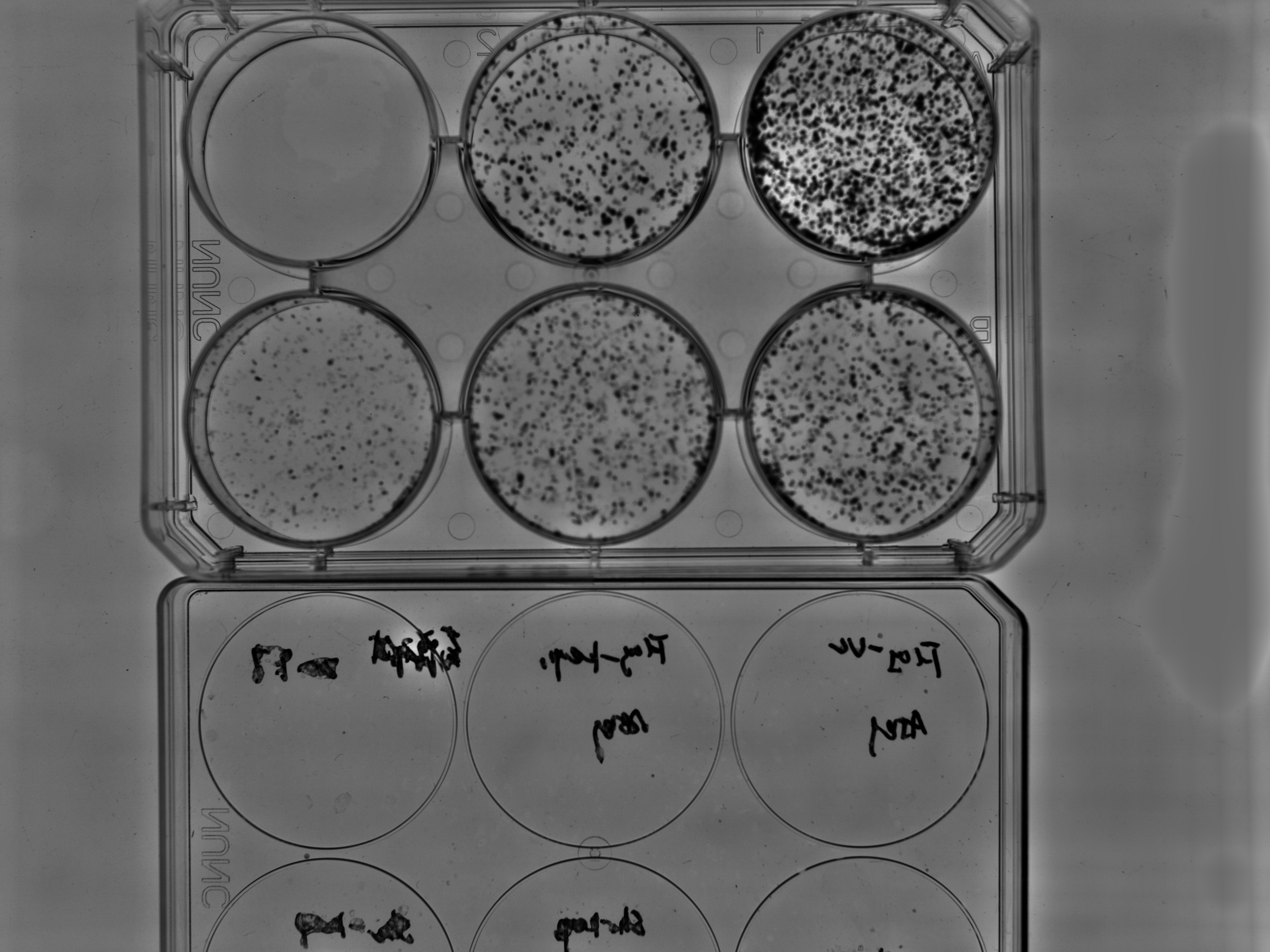

Supplement: Supplemental Information 4 — Cell growth curves and colony formation assays in Fig. 3. [file peerj-09-11908-s004.zip › Figure3 raw data/A549 Colony formation assay.tif]

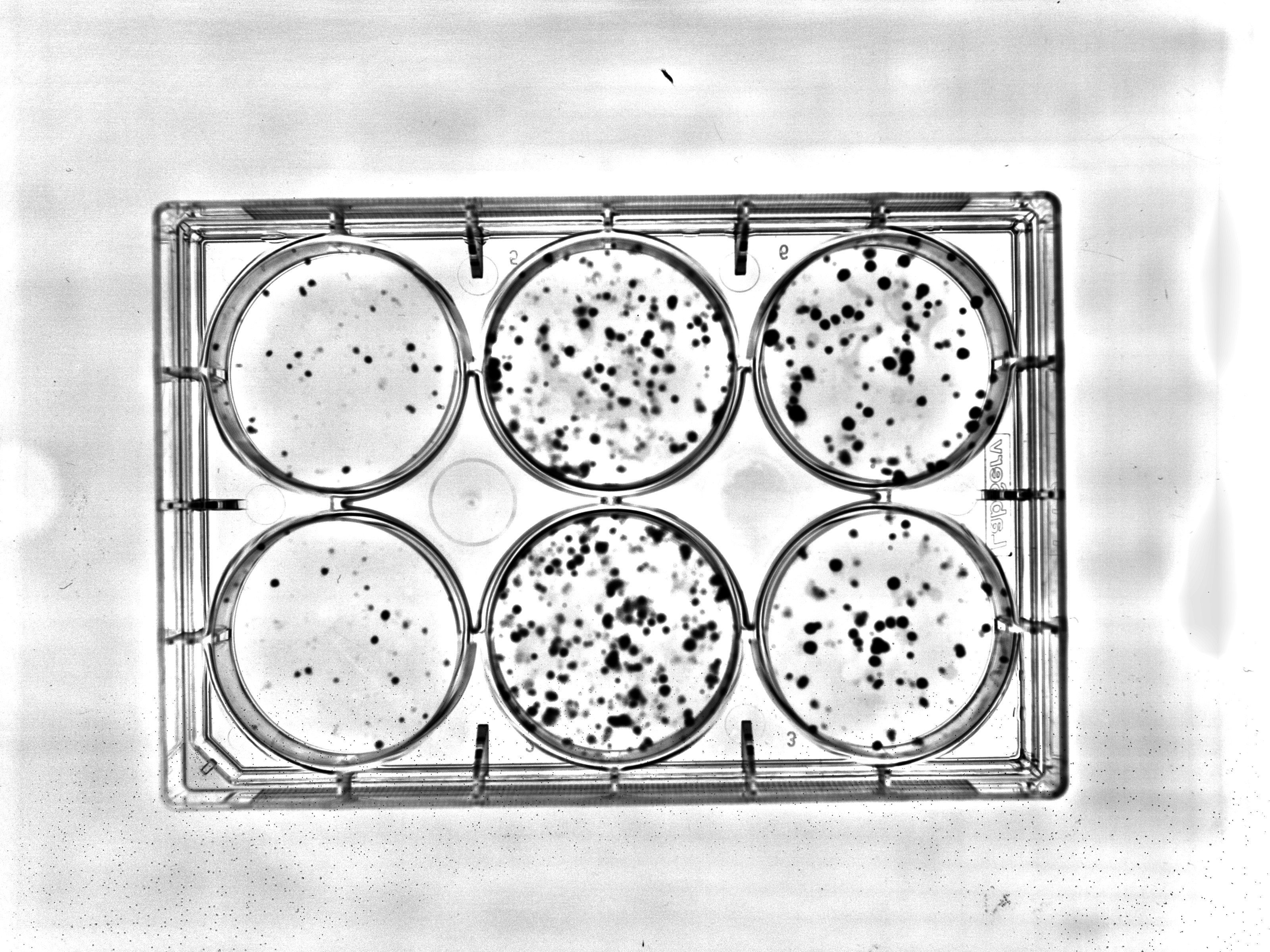

Supplement: Supplemental Information 4 — Cell growth curves and colony formation assays in Fig. 3. [file peerj-09-11908-s004.zip › Figure3 raw data/H1299 Colony formation assay.tif]

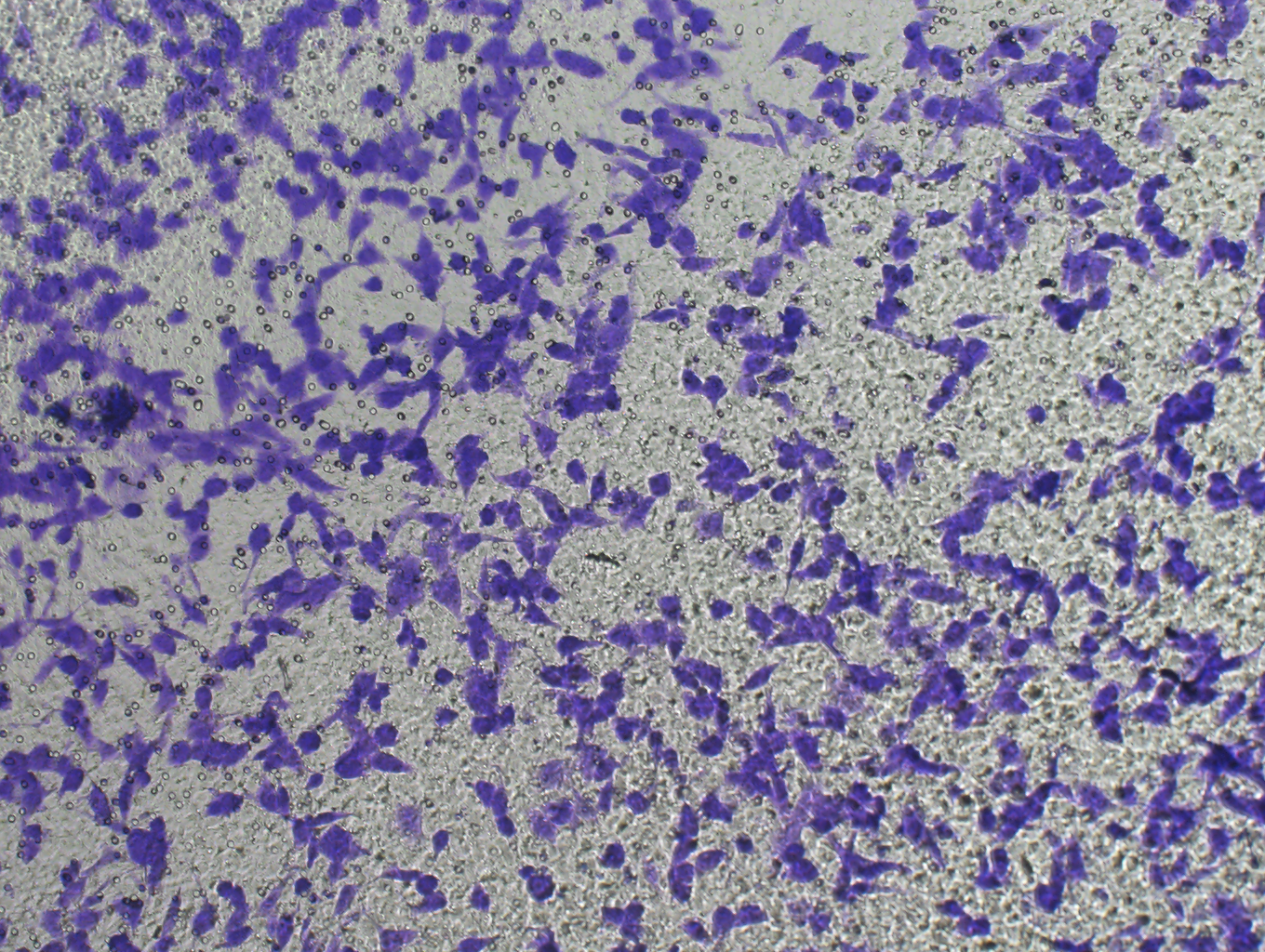

Supplement: Supplemental Information 5 — Transwell invasion assays in Fig. 4. [file peerj-09-11908-s005.zip › Figure4-1 raw data/Transwell invasion assay/Ctrl H1299.tif]

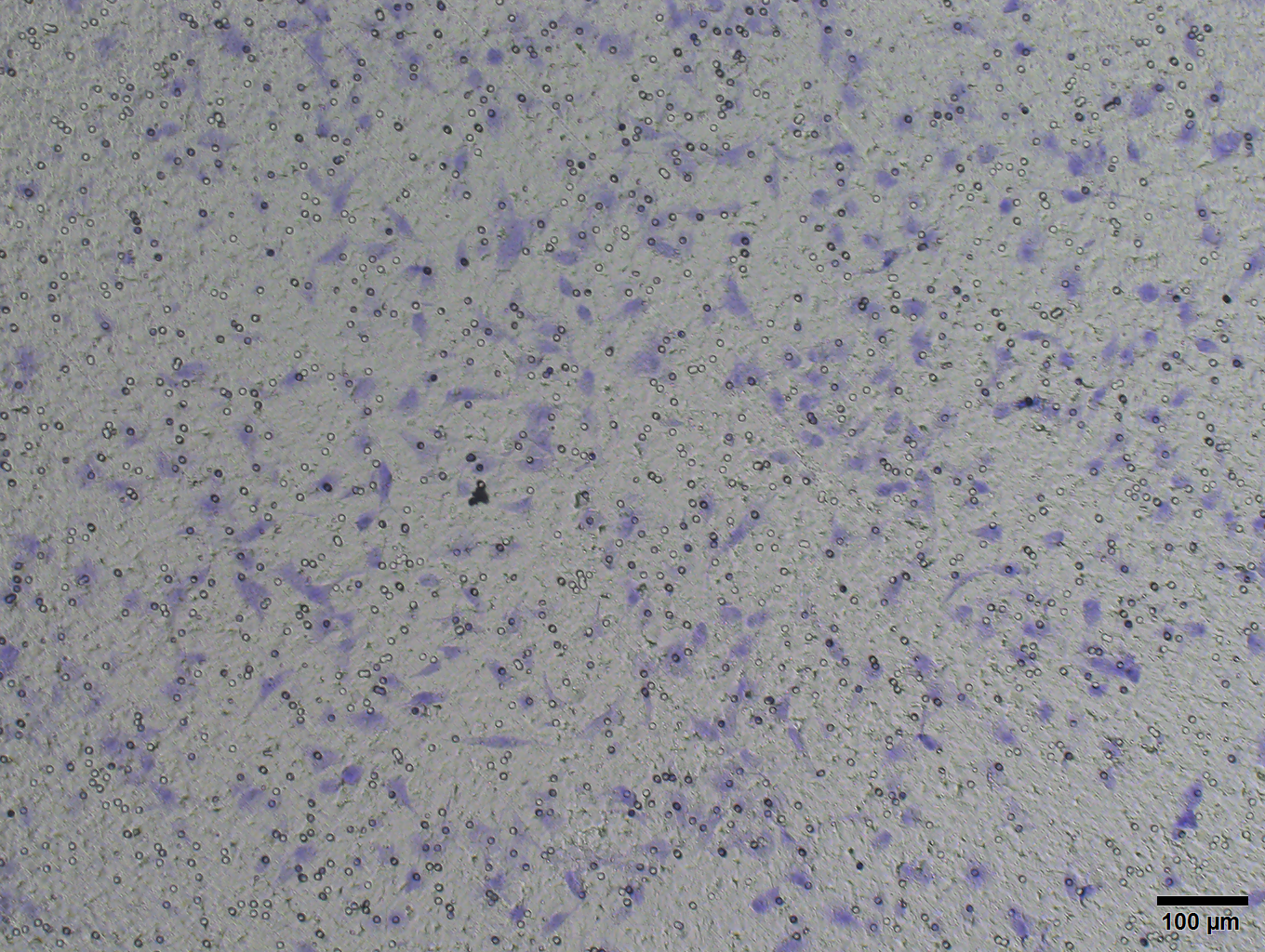

Supplement: Supplemental Information 5 — Transwell invasion assays in Fig. 4. [file peerj-09-11908-s005.zip › Figure4-1 raw data/Transwell invasion assay/Ctrl-A549.tif]

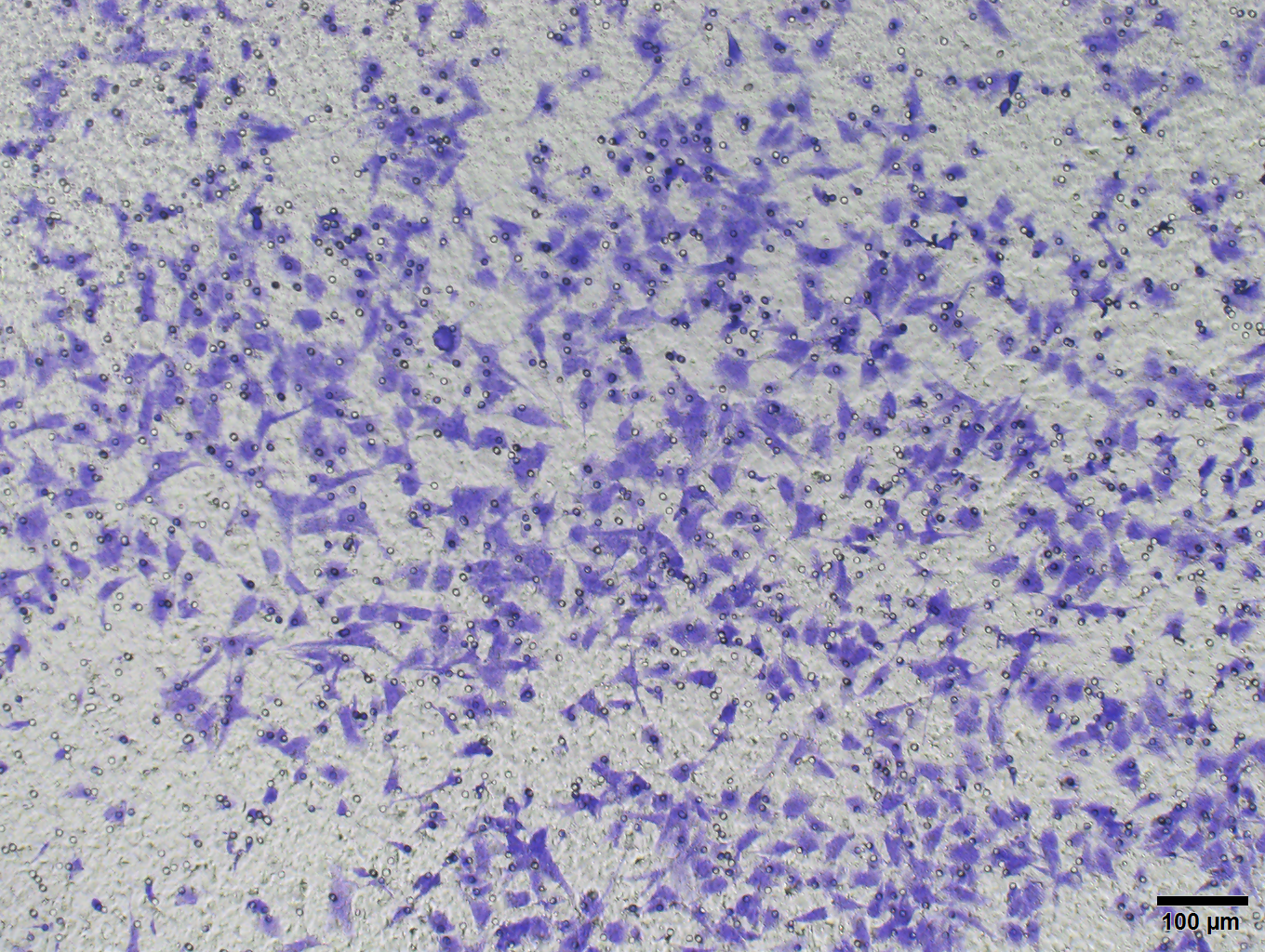

Supplement: Supplemental Information 5 — Transwell invasion assays in Fig. 4. [file peerj-09-11908-s005.zip › Figure4-1 raw data/Transwell invasion assay/KD A549.tif]

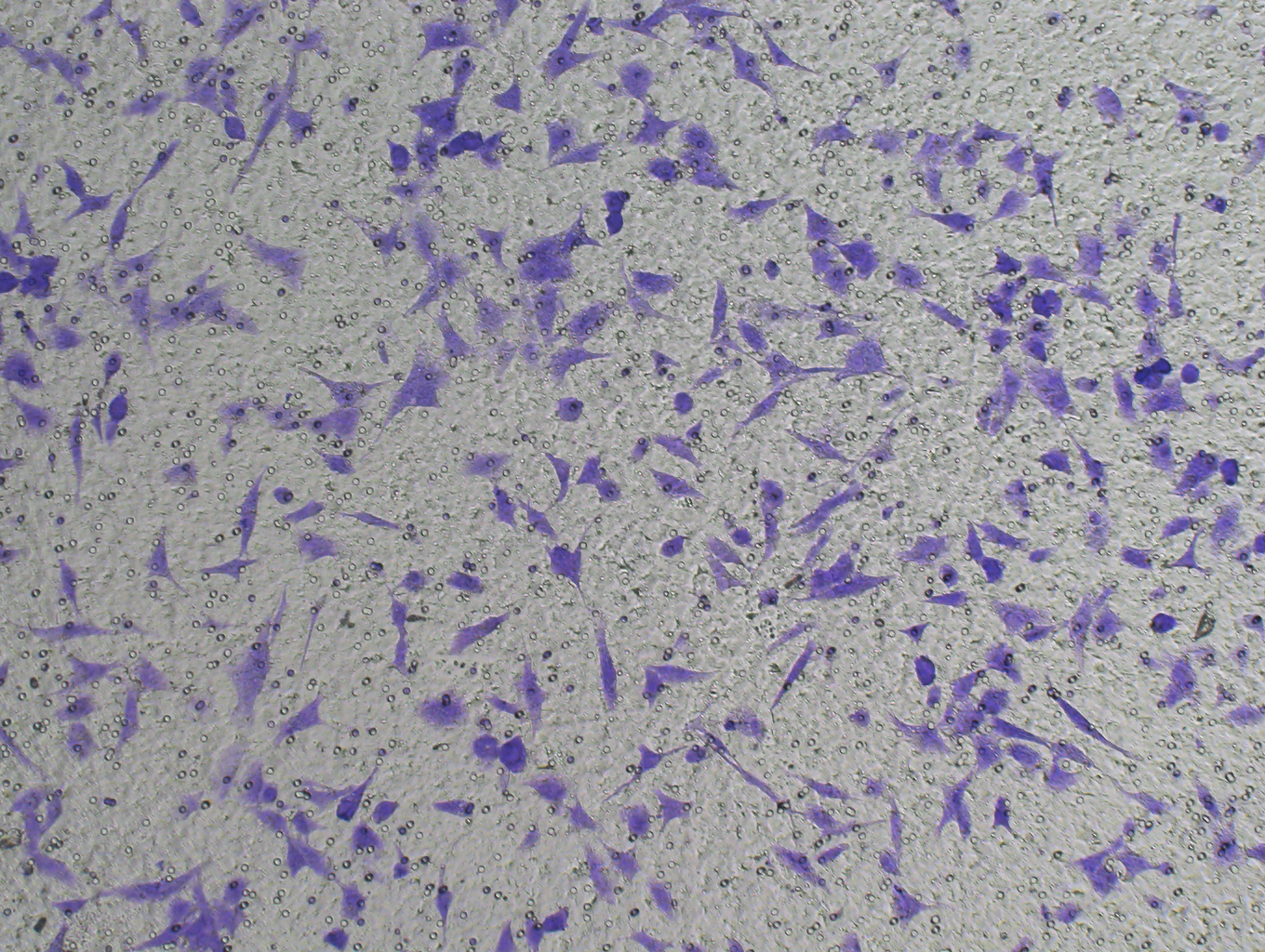

Supplement: Supplemental Information 5 — Transwell invasion assays in Fig. 4. [file peerj-09-11908-s005.zip › Figure4-1 raw data/Transwell invasion assay/OE A549.tif]

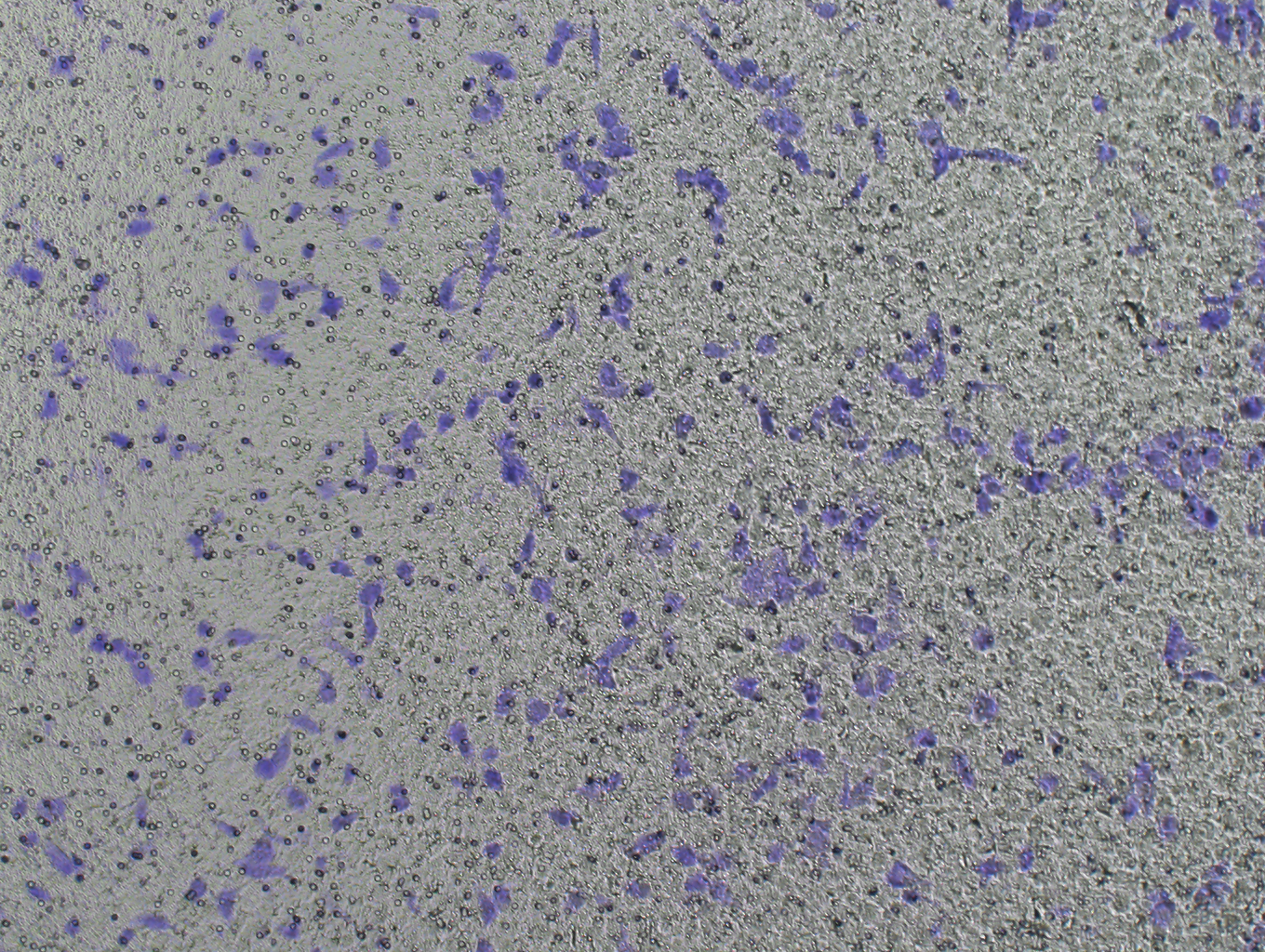

Supplement: Supplemental Information 5 — Transwell invasion assays in Fig. 4. [file peerj-09-11908-s005.zip › Figure4-1 raw data/Transwell invasion assay/OE H1299.tif]

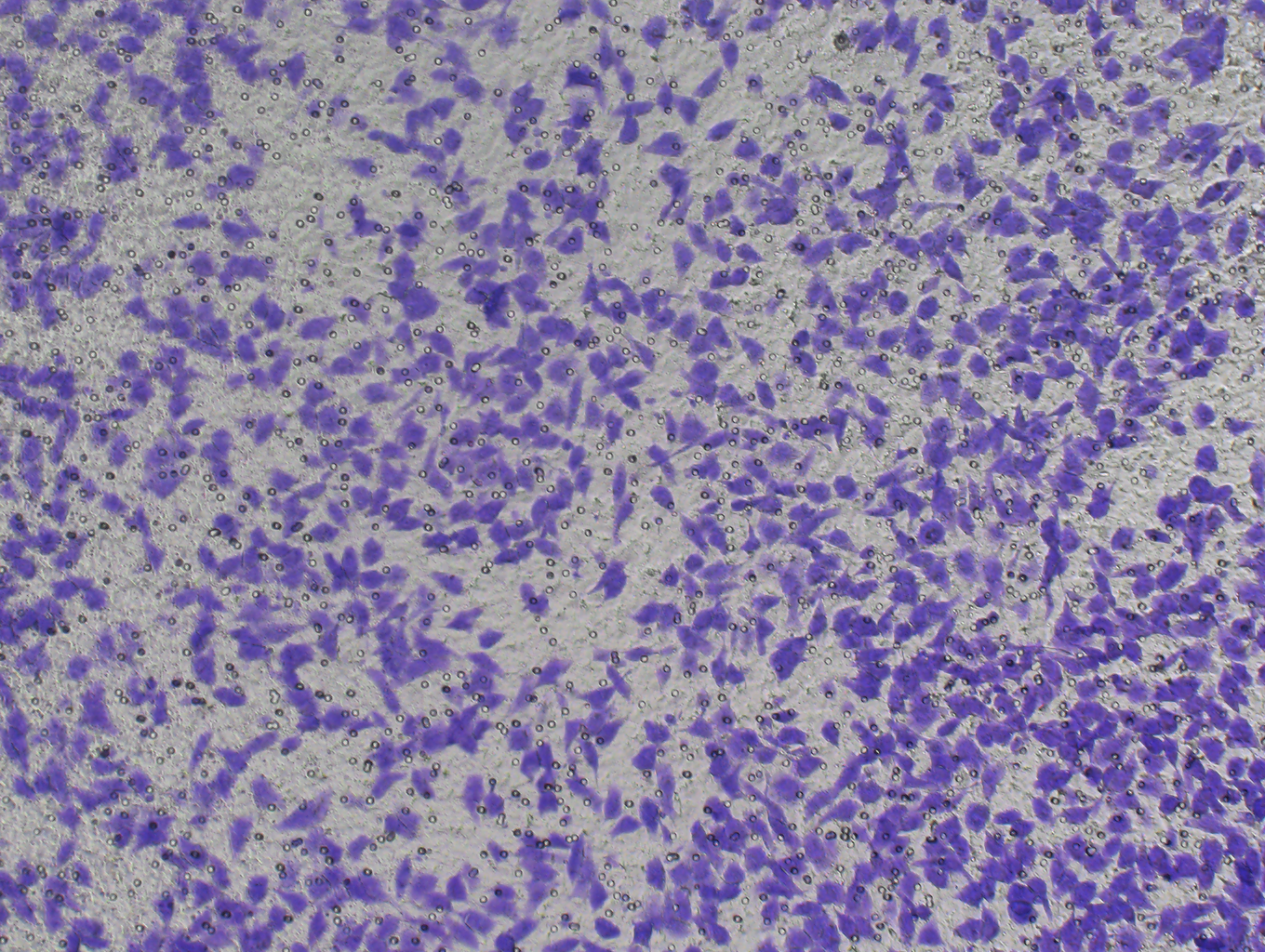

Supplement: Supplemental Information 5 — Transwell invasion assays in Fig. 4. [file peerj-09-11908-s005.zip › Figure4-1 raw data/Transwell invasion assay/SH H1299.tif]

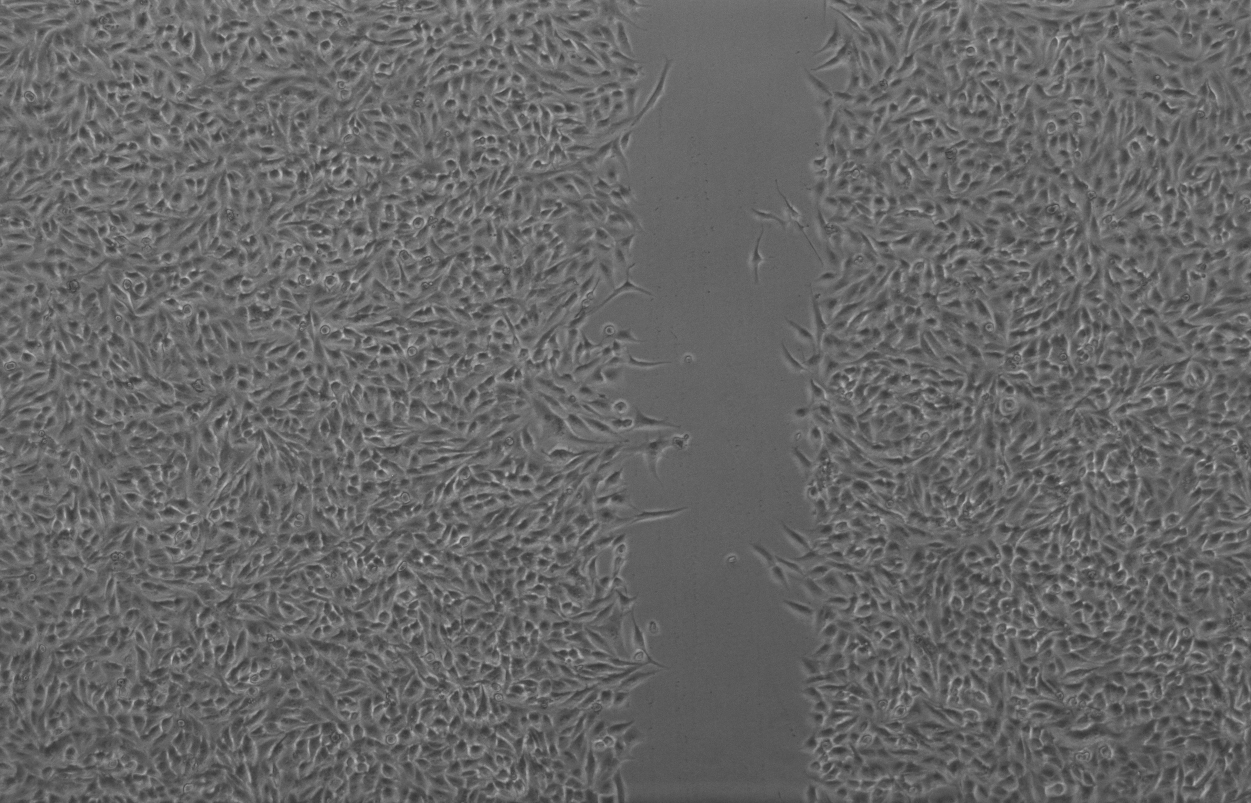

Supplement: Supplemental Information 6 — Wound scratch tests in Fig. 4. [file peerj-09-11908-s006.zip › Figure4-2 raw data/A549 Wound scratch test/Ctrl 24H 20200831.tif]

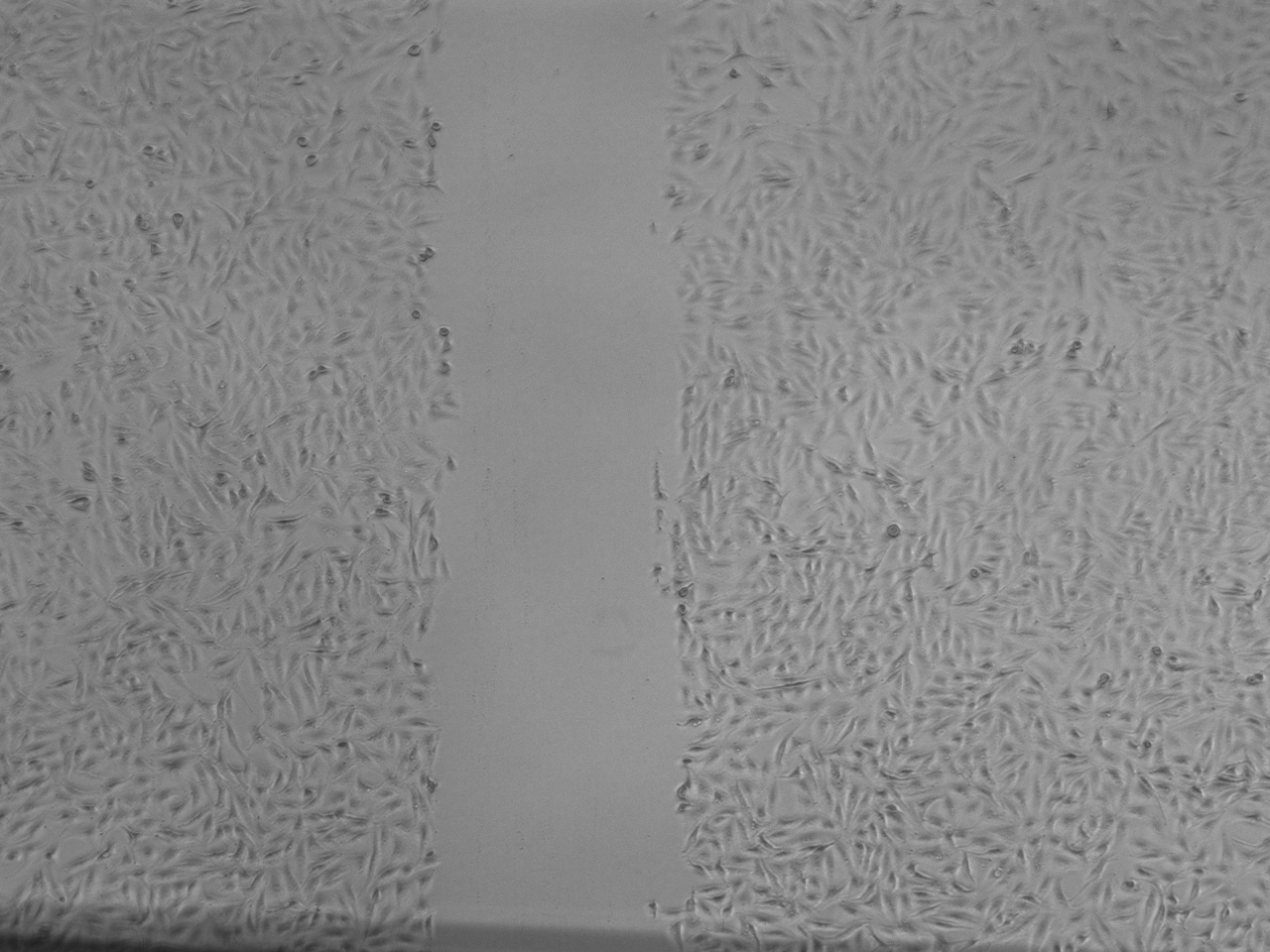

Supplement: Supplemental Information 6 — Wound scratch tests in Fig. 4. [file peerj-09-11908-s006.zip › Figure4-2 raw data/A549 Wound scratch test/Ctrl 0H 20200830.tif]

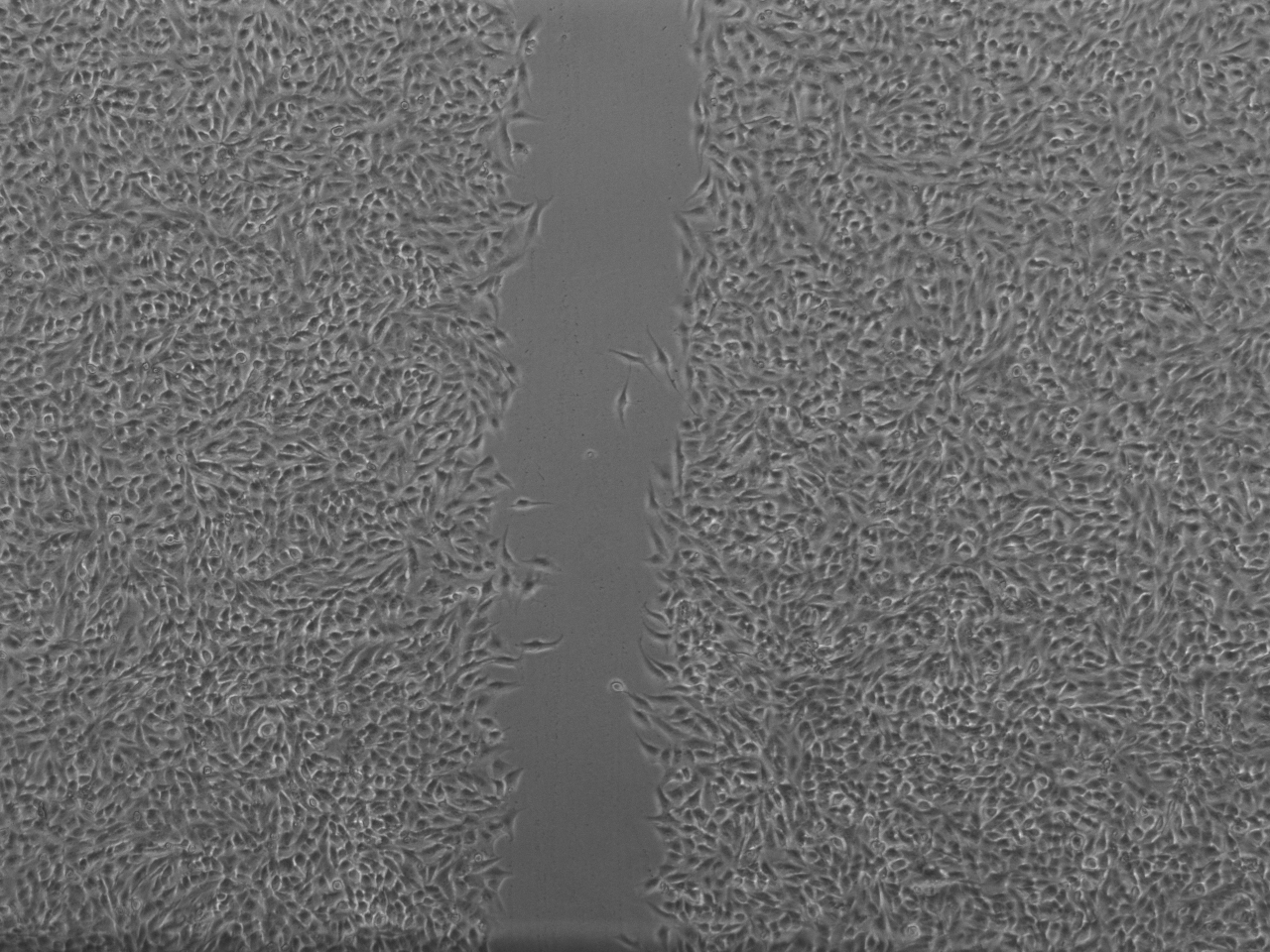

Supplement: Supplemental Information 6 — Wound scratch tests in Fig. 4. [file peerj-09-11908-s006.zip › Figure4-2 raw data/A549 Wound scratch test/Ctrl 48h 20200901.tif]

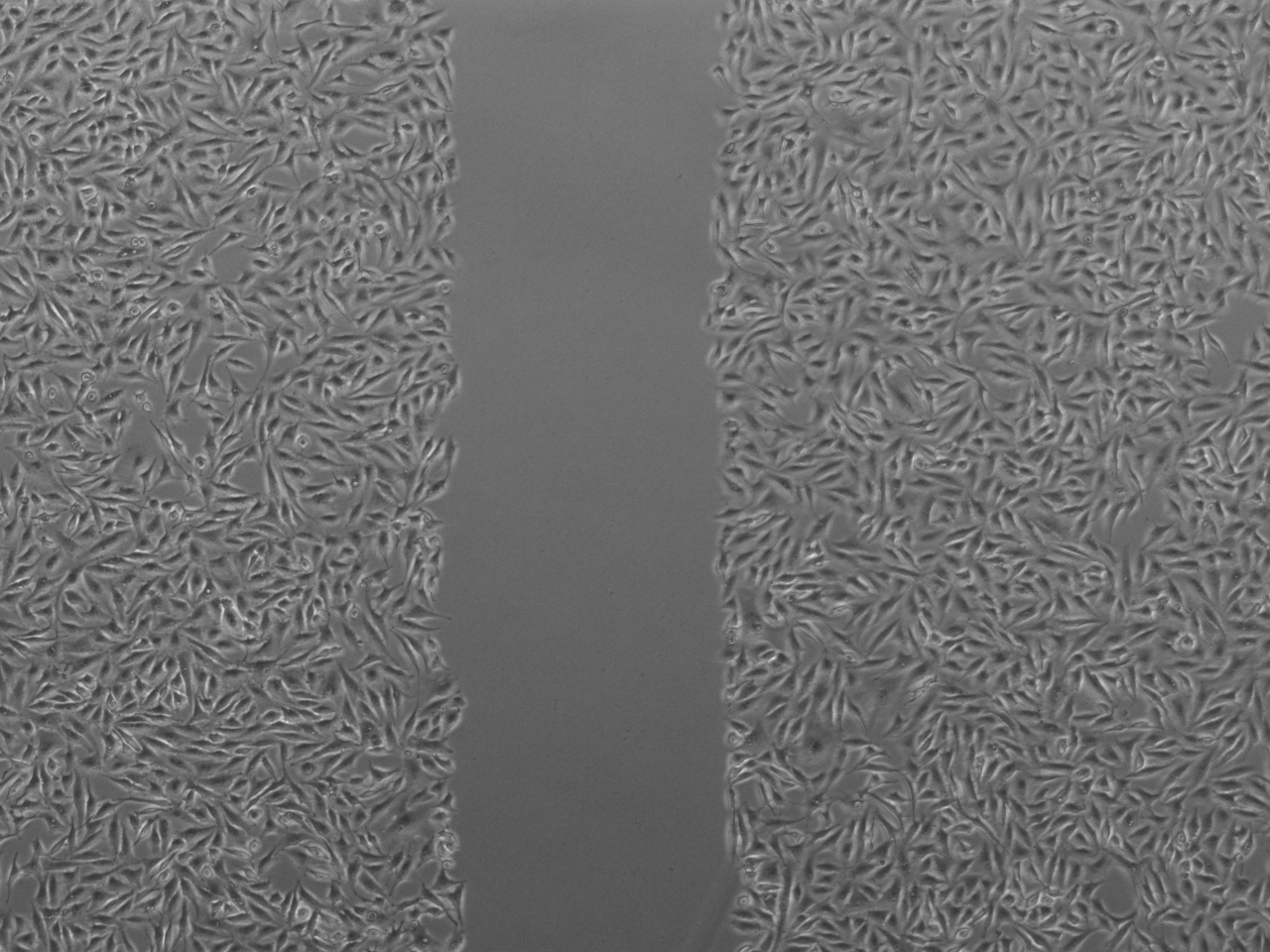

Supplement: Supplemental Information 6 — Wound scratch tests in Fig. 4. [file peerj-09-11908-s006.zip › Figure4-2 raw data/A549 Wound scratch test/KD 0H 20200830.tif]

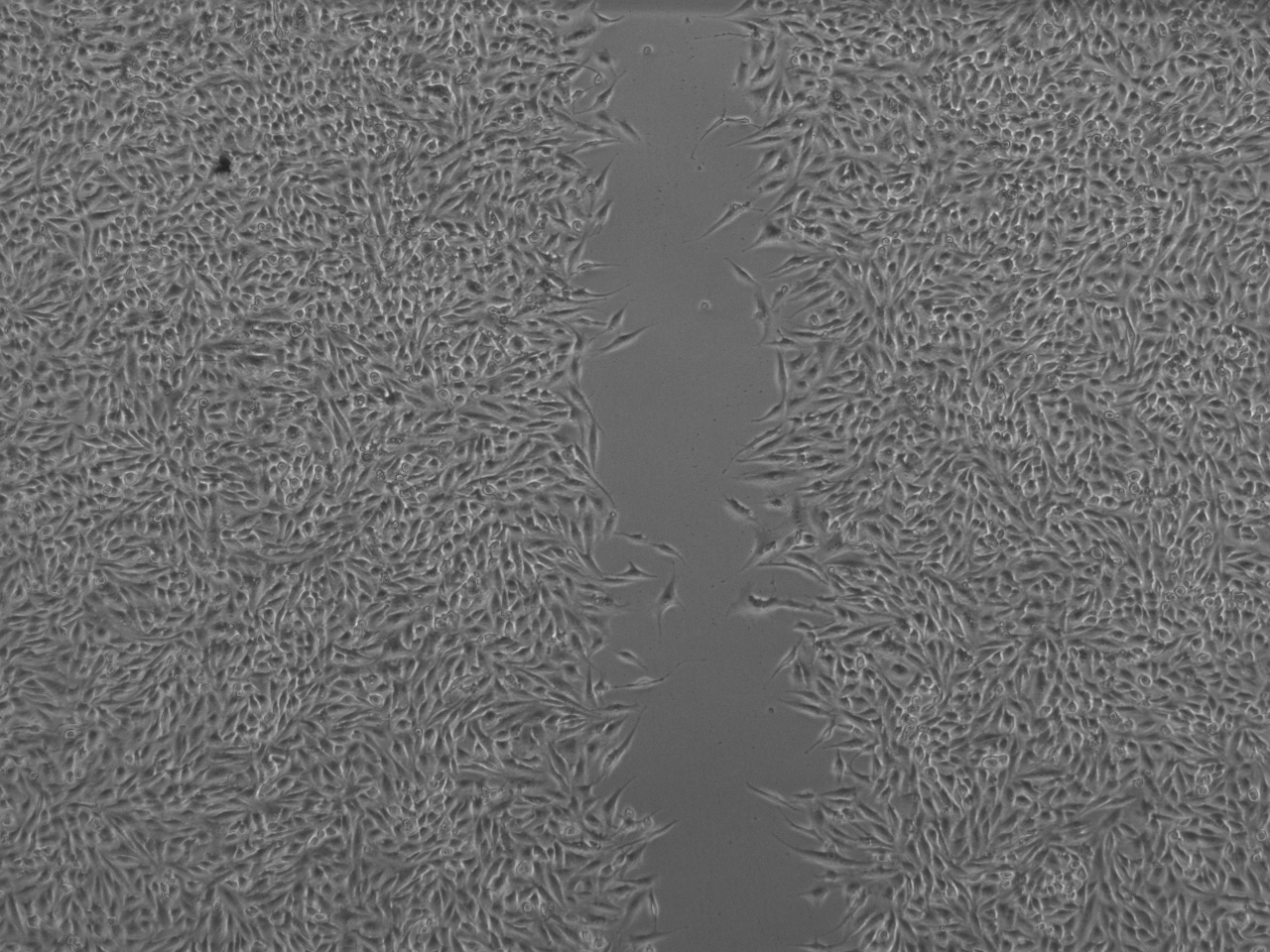

Supplement: Supplemental Information 6 — Wound scratch tests in Fig. 4. [file peerj-09-11908-s006.zip › Figure4-2 raw data/A549 Wound scratch test/KD 24H 20200831.tif]

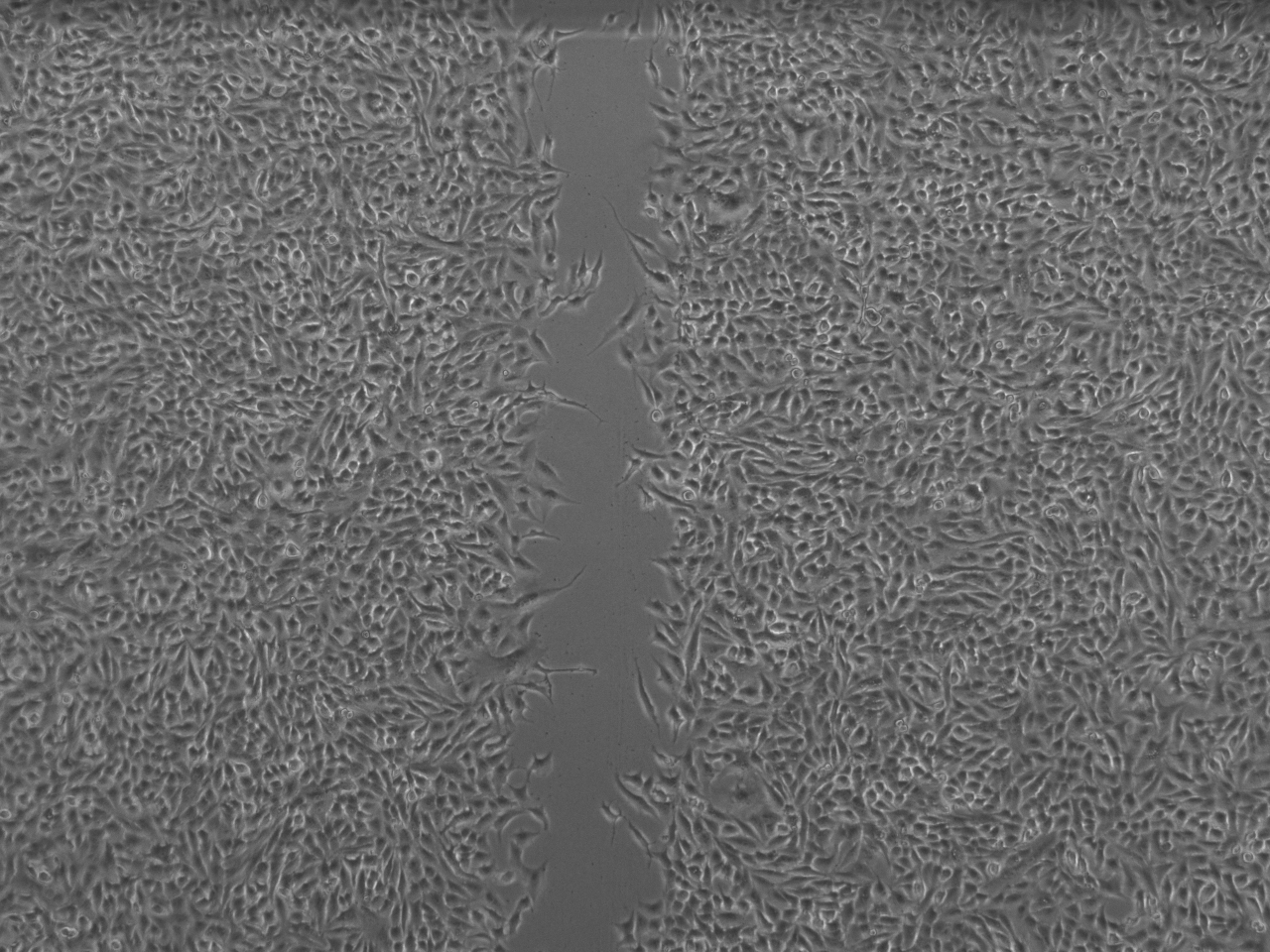

Supplement: Supplemental Information 6 — Wound scratch tests in Fig. 4. [file peerj-09-11908-s006.zip › Figure4-2 raw data/A549 Wound scratch test/kD 48H 20200901.tif]

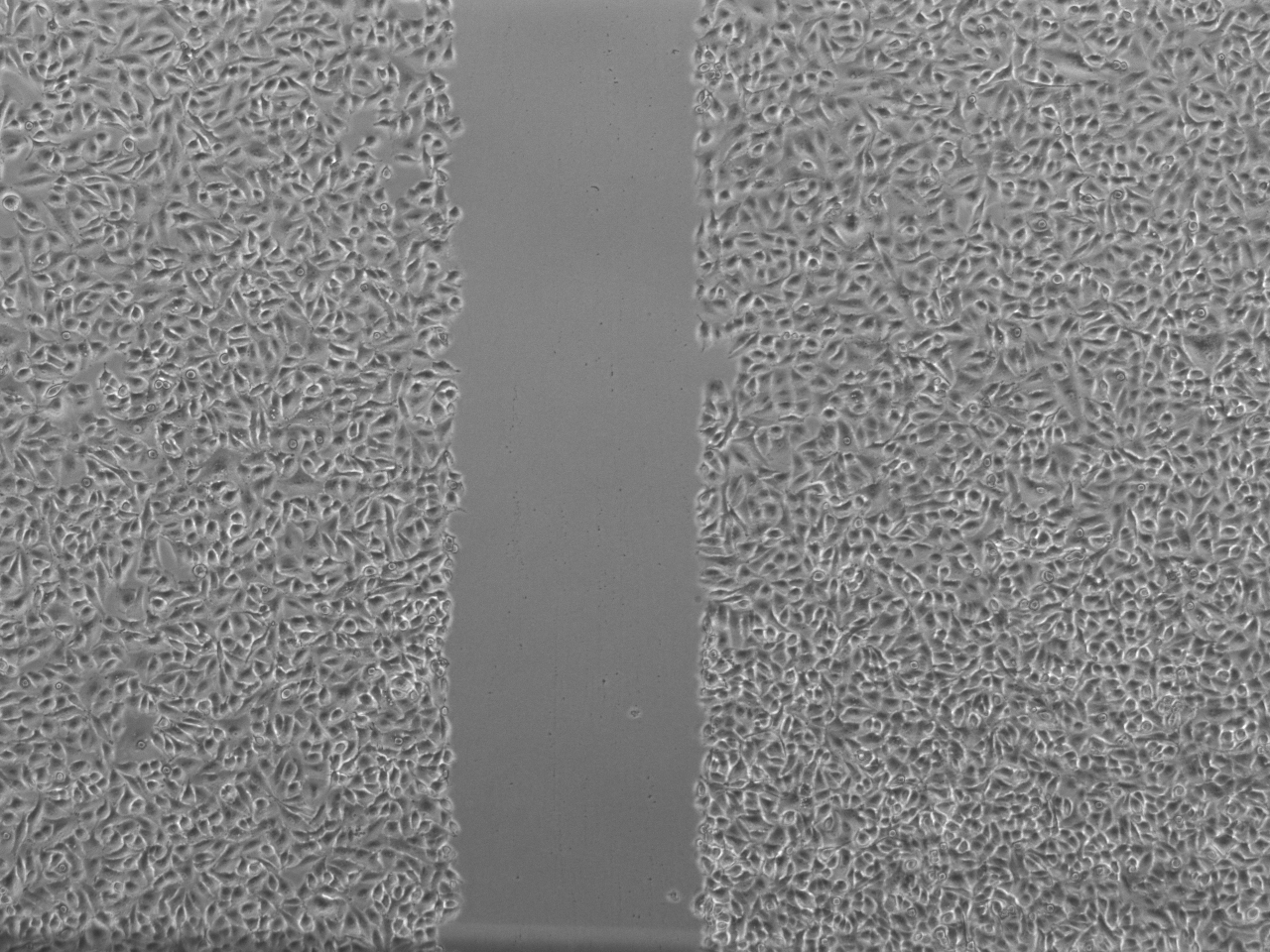

Supplement: Supplemental Information 6 — Wound scratch tests in Fig. 4. [file peerj-09-11908-s006.zip › Figure4-2 raw data/A549 Wound scratch test/OE 0H 20200830.tif]

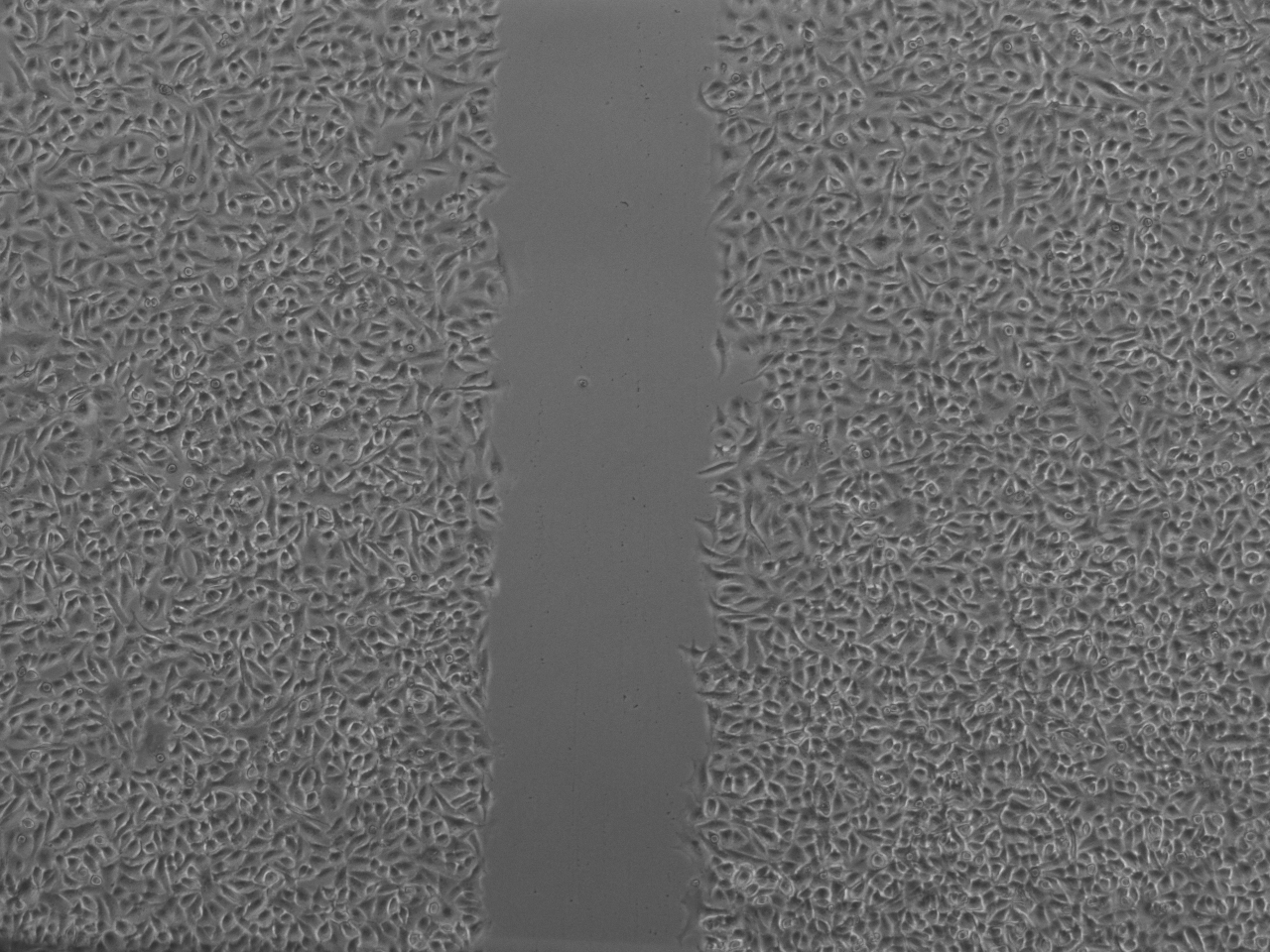

Supplement: Supplemental Information 6 — Wound scratch tests in Fig. 4. [file peerj-09-11908-s006.zip › Figure4-2 raw data/A549 Wound scratch test/OE 24H 20200831.tif]

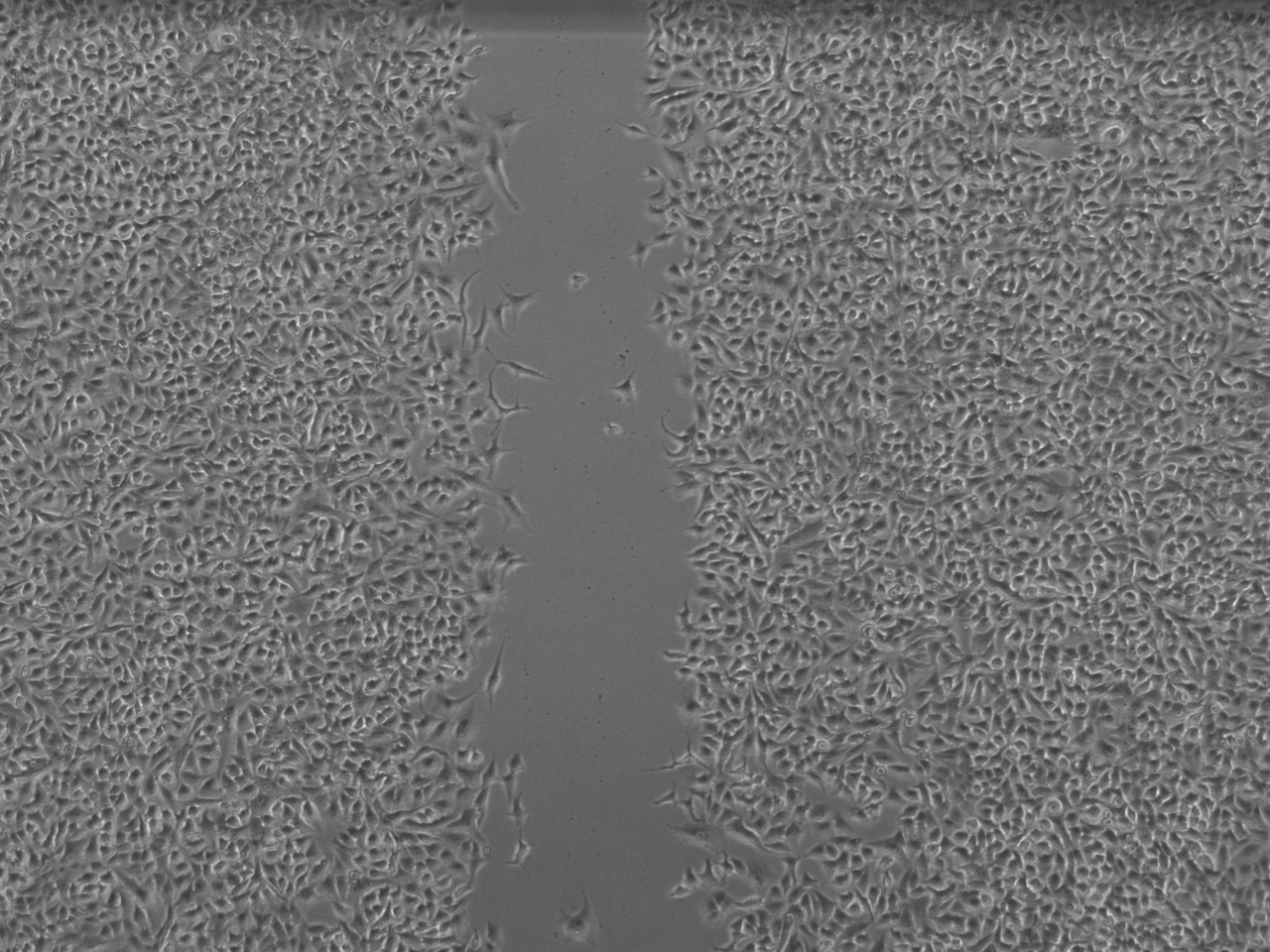

Supplement: Supplemental Information 6 — Wound scratch tests in Fig. 4. [file peerj-09-11908-s006.zip › Figure4-2 raw data/A549 Wound scratch test/OE 48H 20200901.tif]

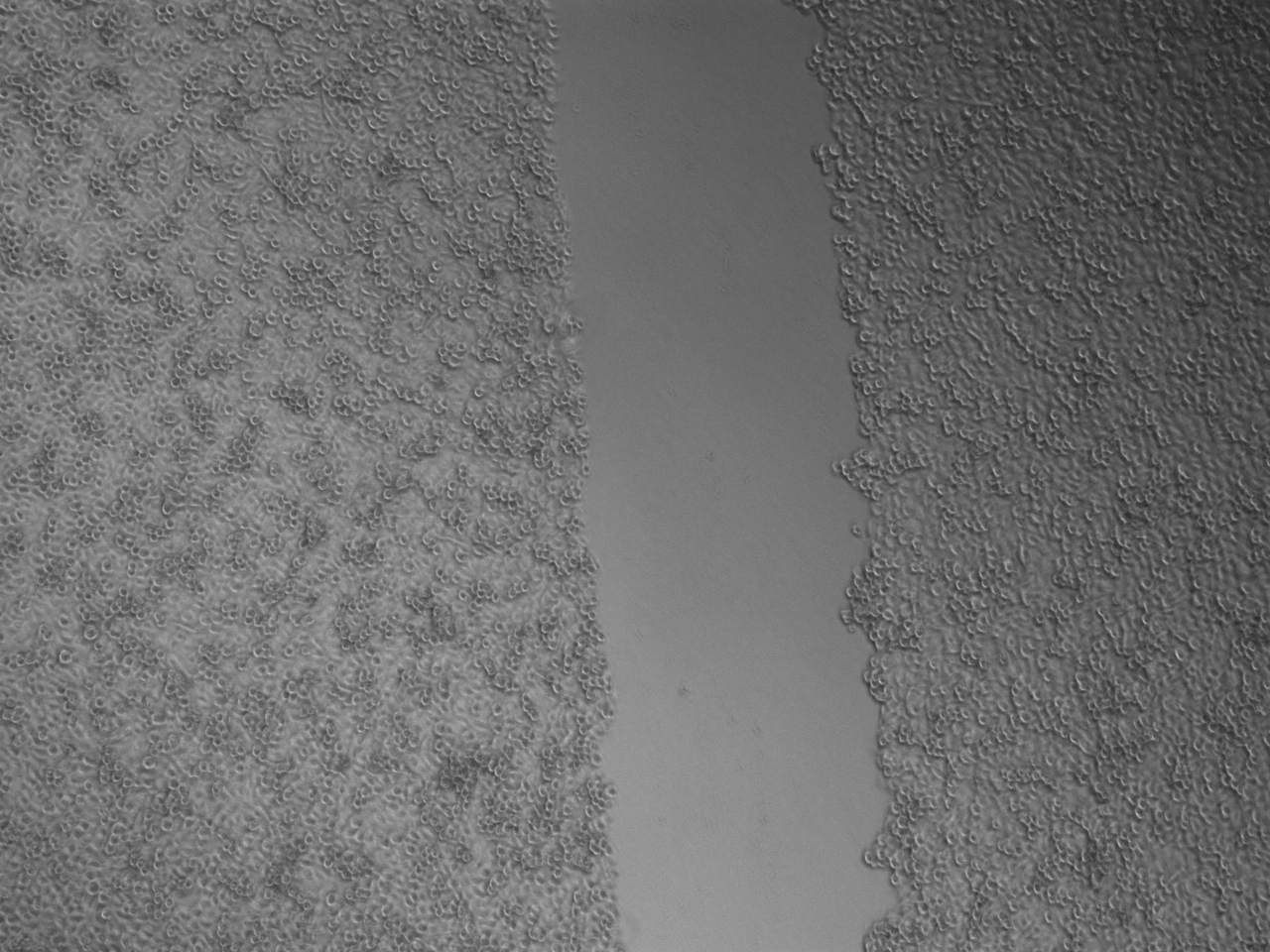

Supplement: Supplemental Information 6 — Wound scratch tests in Fig. 4. [file peerj-09-11908-s006.zip › Figure4-2 raw data/H1299 Wound scratch test/Ctrl 0H.tif]

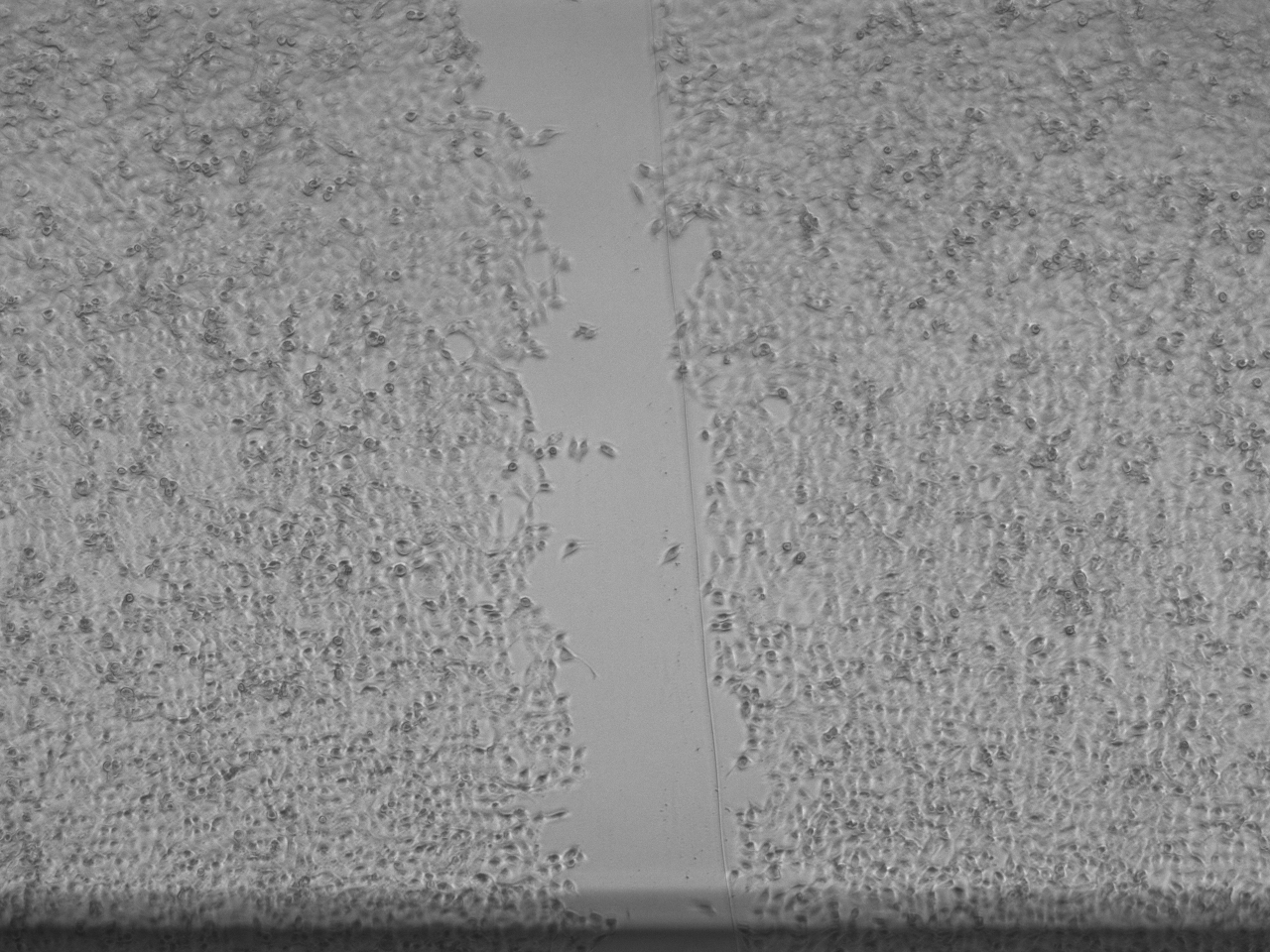

Supplement: Supplemental Information 6 — Wound scratch tests in Fig. 4. [file peerj-09-11908-s006.zip › Figure4-2 raw data/H1299 Wound scratch test/Ctrl 12H.tif]

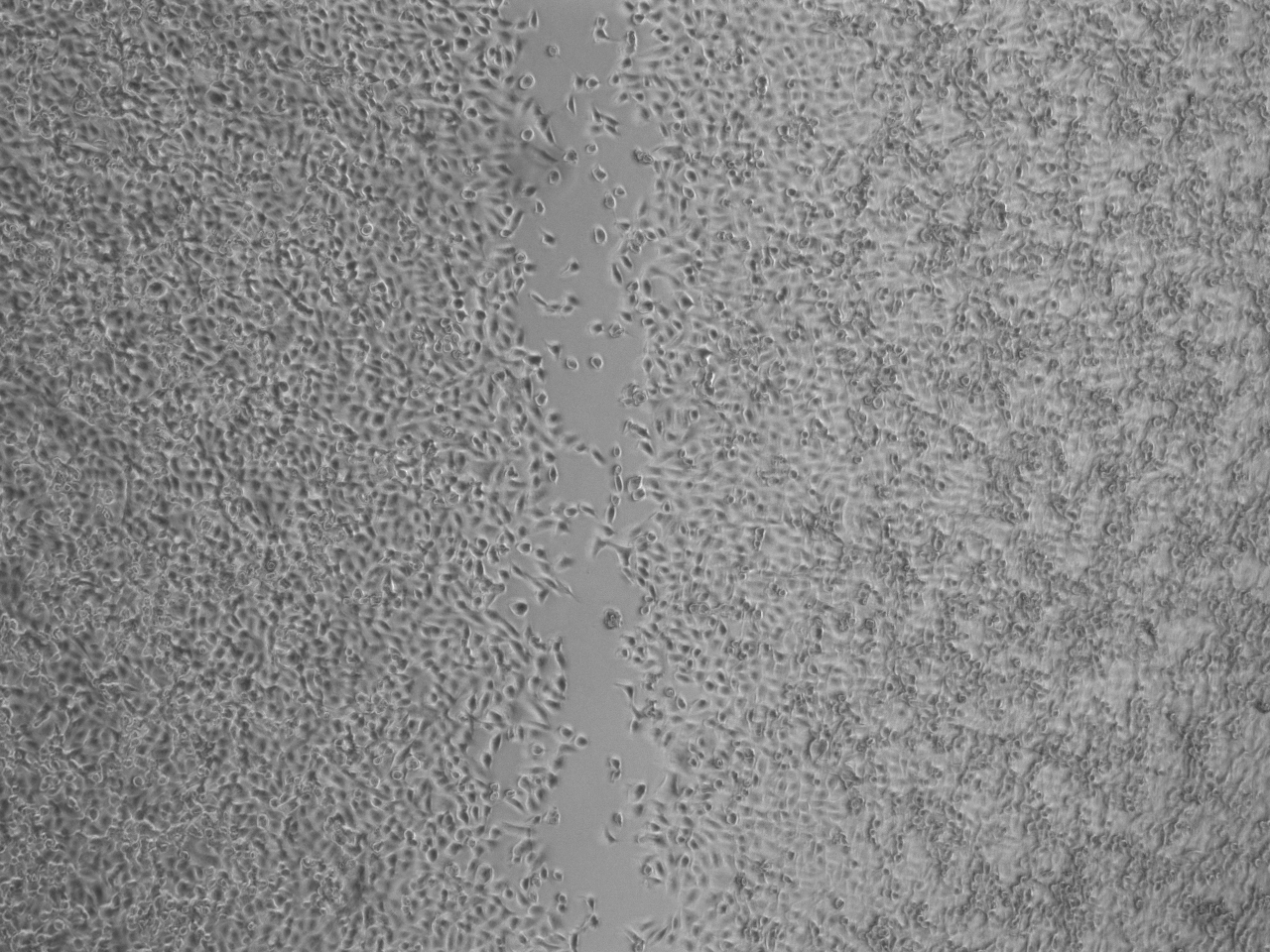

Supplement: Supplemental Information 6 — Wound scratch tests in Fig. 4. [file peerj-09-11908-s006.zip › Figure4-2 raw data/H1299 Wound scratch test/Ctrl 24H.tif]

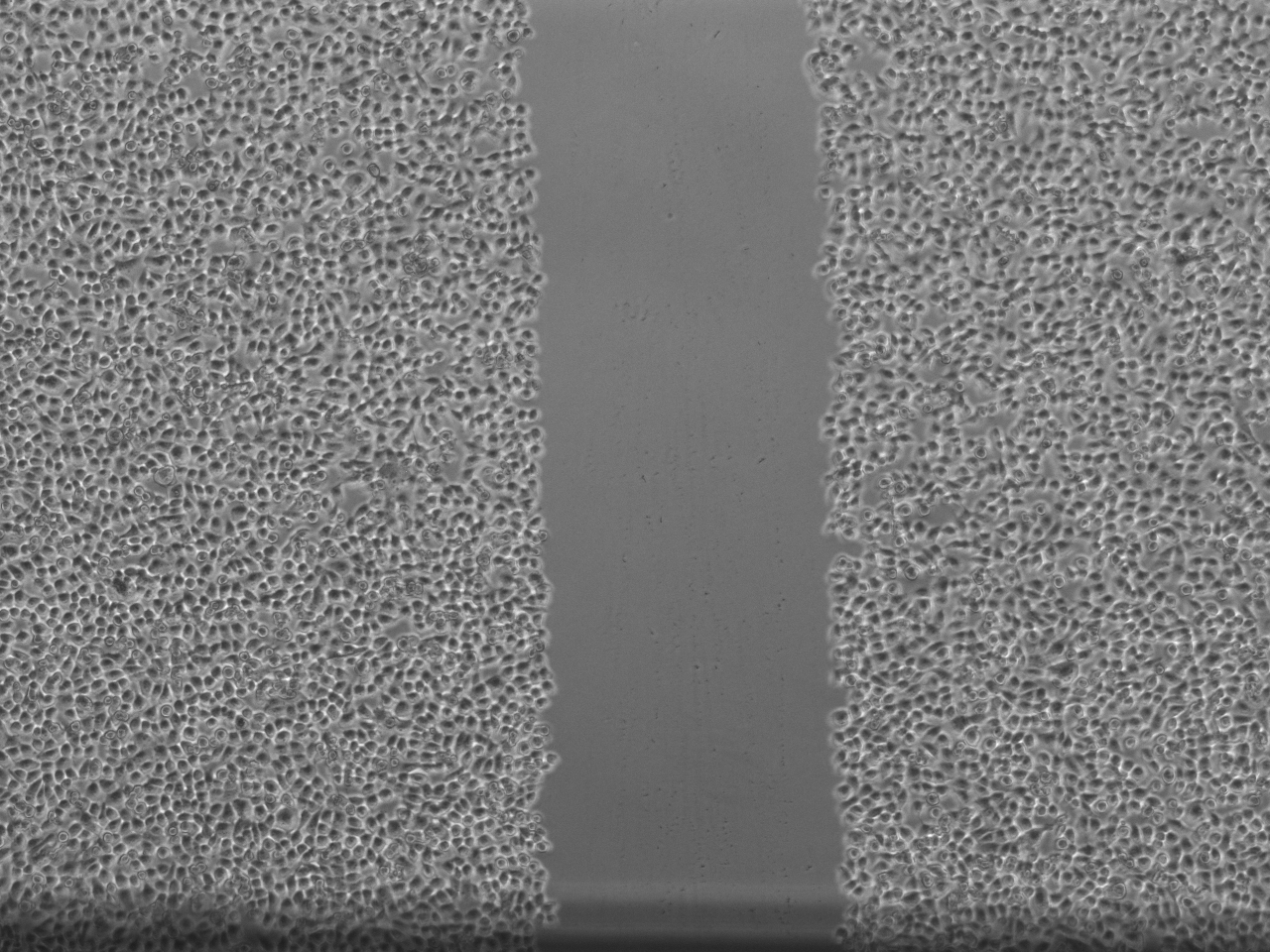

Supplement: Supplemental Information 6 — Wound scratch tests in Fig. 4. [file peerj-09-11908-s006.zip › Figure4-2 raw data/H1299 Wound scratch test/KD 0H.tif]

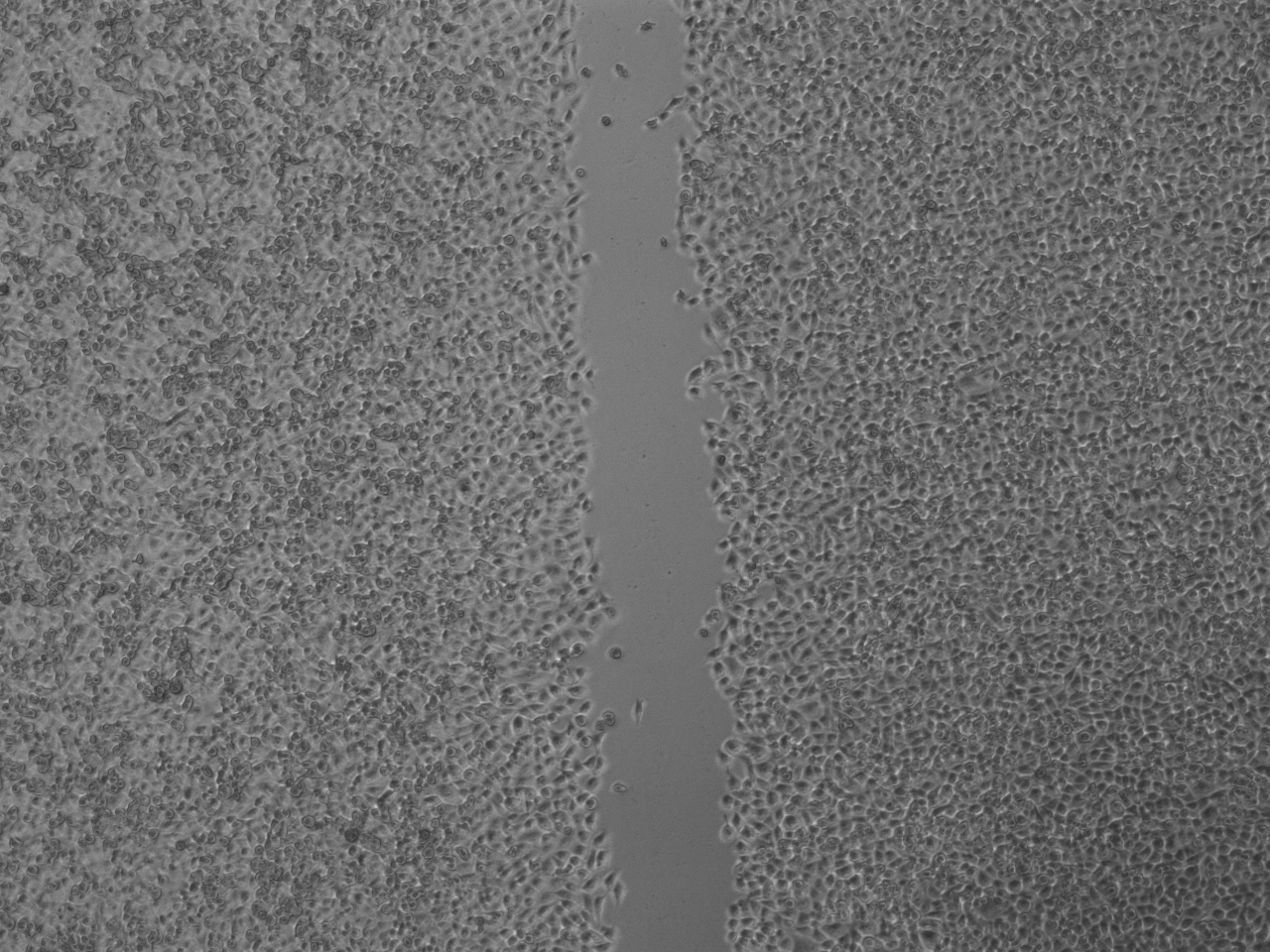

Supplement: Supplemental Information 6 — Wound scratch tests in Fig. 4. [file peerj-09-11908-s006.zip › Figure4-2 raw data/H1299 Wound scratch test/KD 12H.tif]

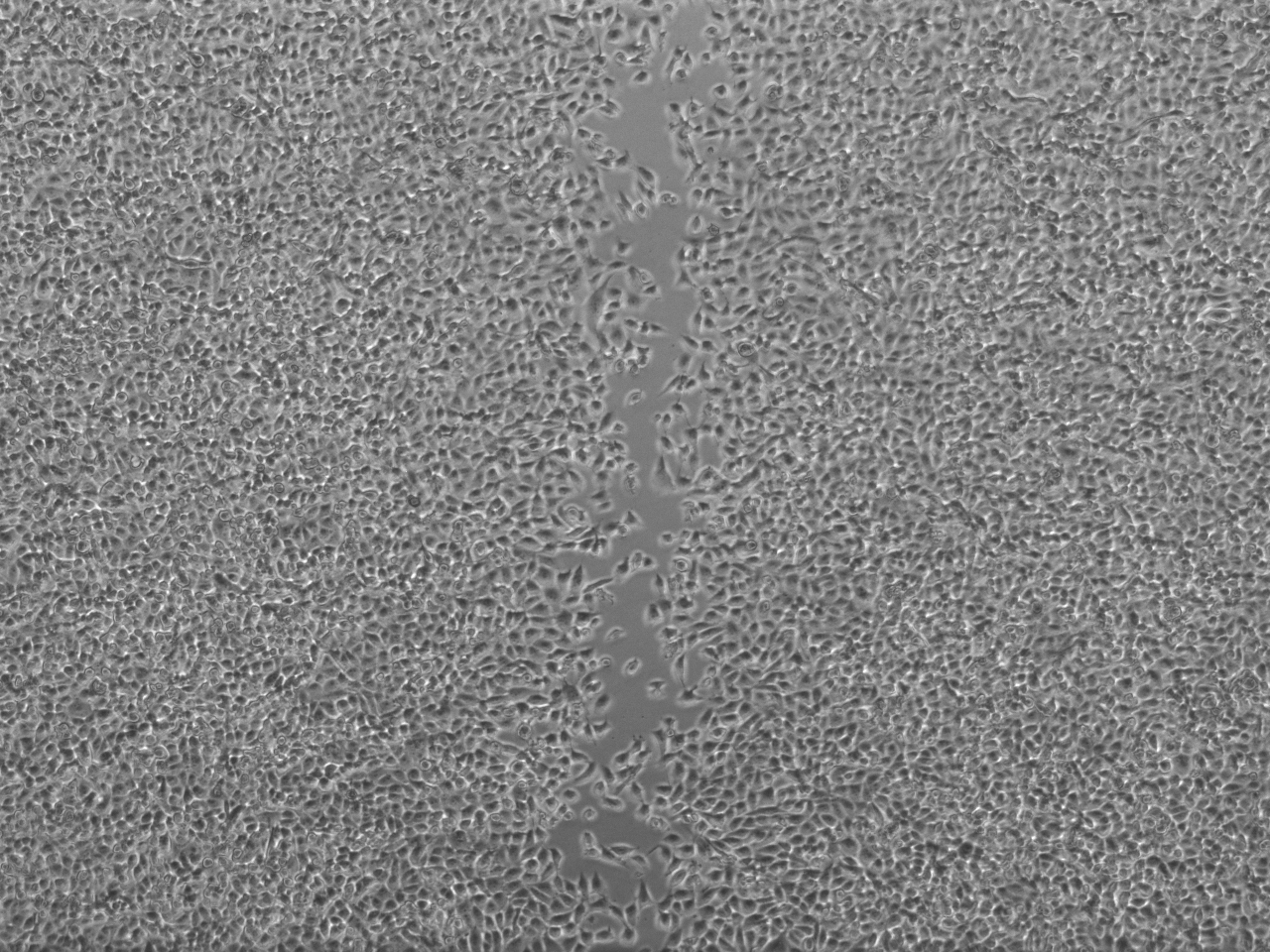

Supplement: Supplemental Information 6 — Wound scratch tests in Fig. 4. [file peerj-09-11908-s006.zip › Figure4-2 raw data/H1299 Wound scratch test/KD 24H.tif]

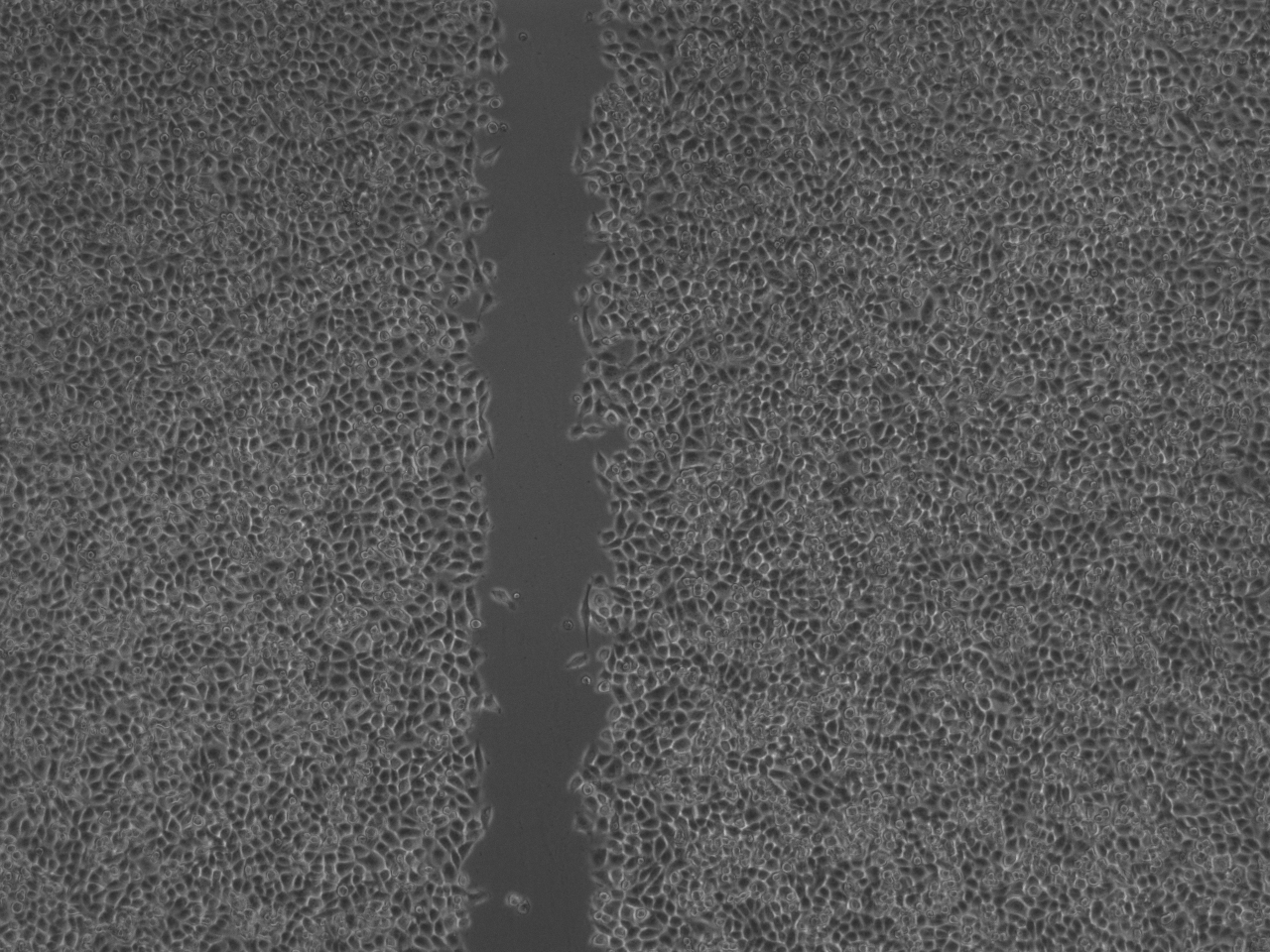

Supplement: Supplemental Information 6 — Wound scratch tests in Fig. 4. [file peerj-09-11908-s006.zip › Figure4-2 raw data/H1299 Wound scratch test/OE 24H.tif]

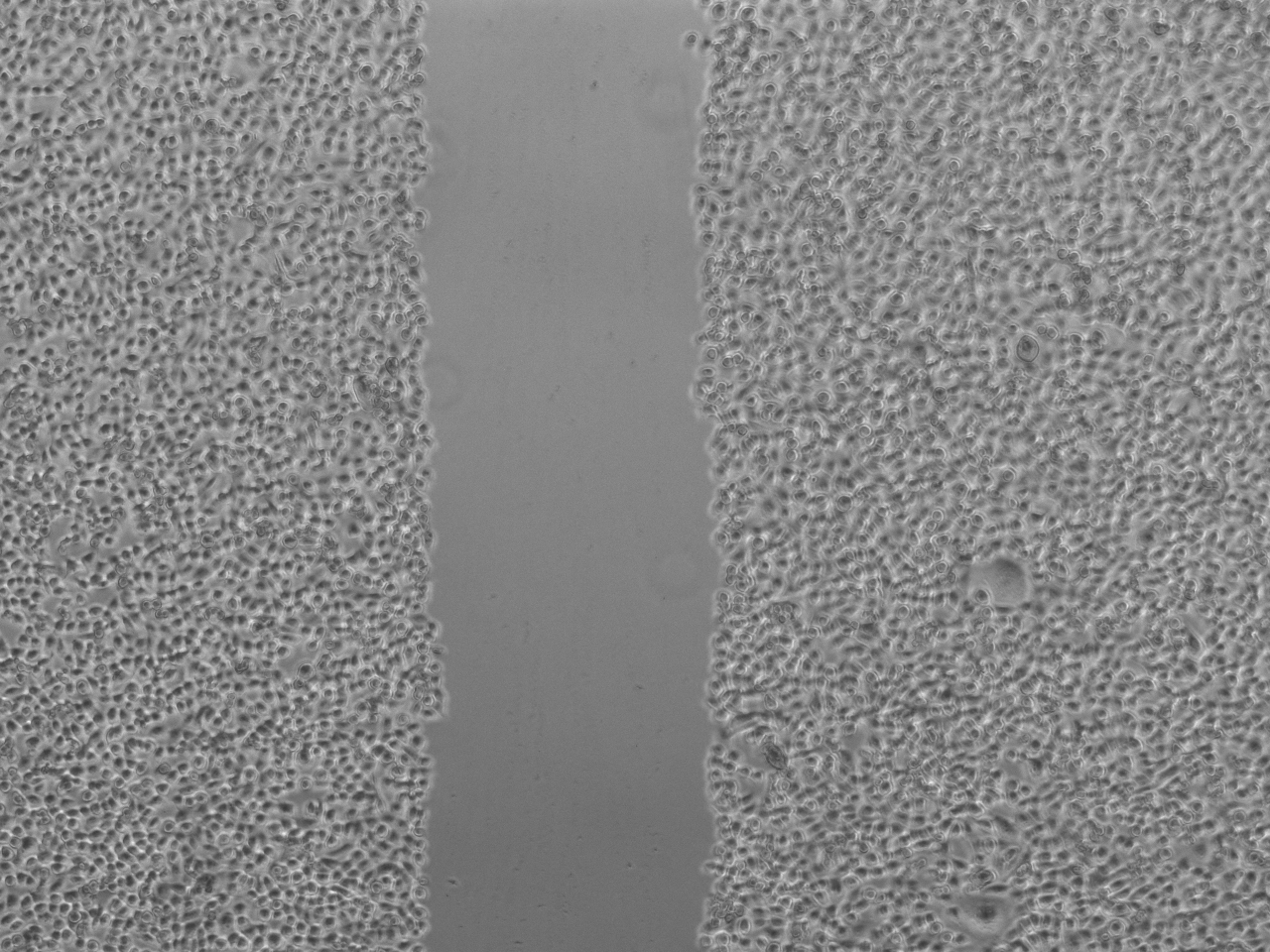

Supplement: Supplemental Information 6 — Wound scratch tests in Fig. 4. [file peerj-09-11908-s006.zip › Figure4-2 raw data/H1299 Wound scratch test/OE 0H.tif]

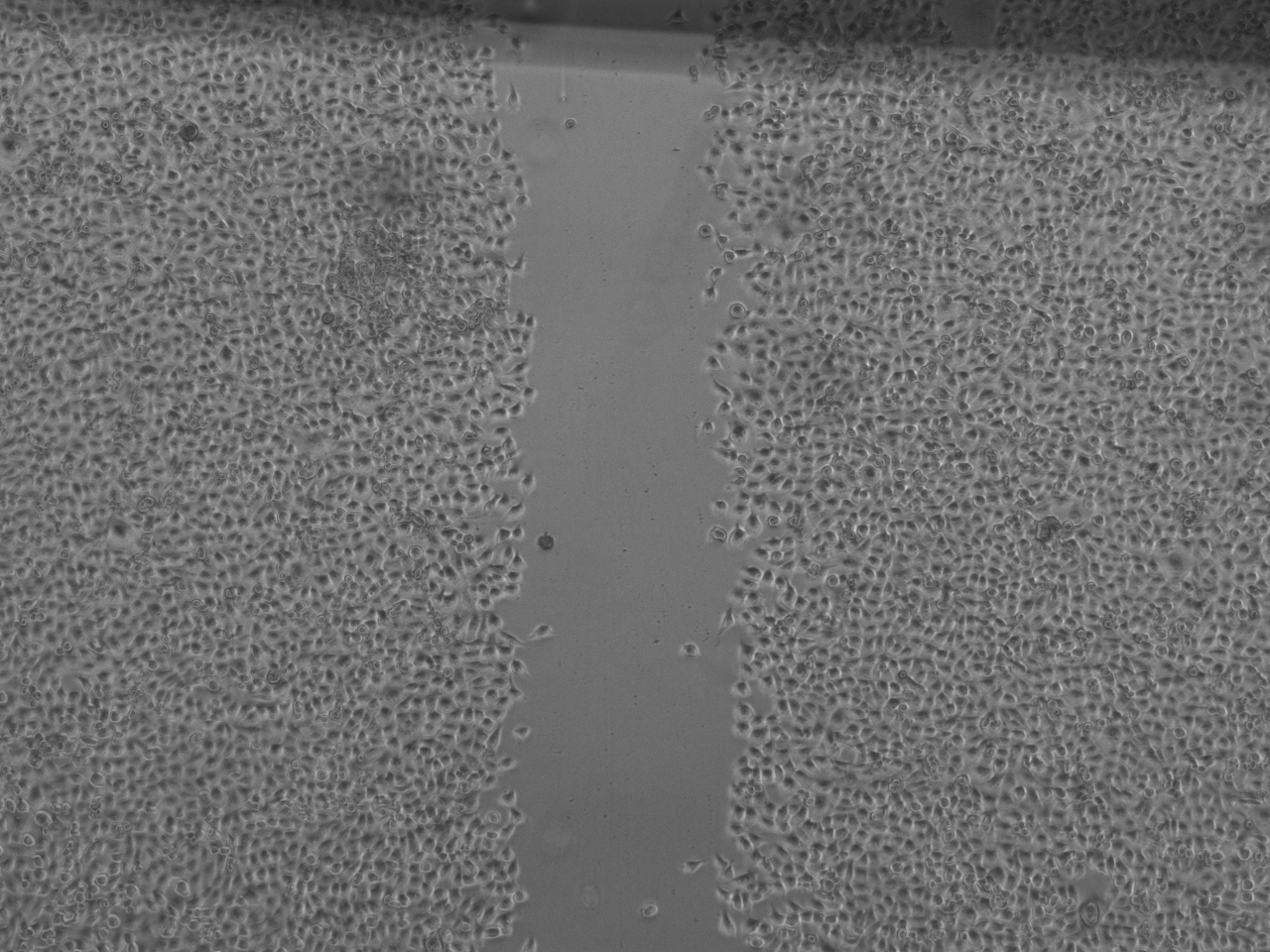

Supplement: Supplemental Information 6 — Wound scratch tests in Fig. 4. [file peerj-09-11908-s006.zip › Figure4-2 raw data/H1299 Wound scratch test/OE12H.tif]
